# The *Caenorhabditis elegans* JNK Signaling Pathway Activates Expression of Stress Response Genes by Derepressing the Fos/HDAC Repressor Complex

**DOI:** 10.1371/journal.pgen.1003315

**Published:** 2013-02-21

**Authors:** Ayuna Hattori, Tomoaki Mizuno, Mayuko Akamatsu, Naoki Hisamoto, Kunihiro Matsumoto

**Affiliations:** 1 Department of Molecular Biology, Graduate School of Science, Nagoya University, Nagoya, Japan; 2 Department of Molecular Cell Biology, Graduate School of Comprehensive Human Sciences, University of Tsukuba, Tsukuba, Japan; The University of Texas Health Science Center at Houston, United States of America

## Abstract

MAP kinases are integral to the mechanisms by which cells respond to a wide variety of environmental stresses. In *Caenorhabditis elegans*, the KGB-1 JNK signaling pathway regulates the response to heavy metal stress. In this study, we identified FOS-1, a bZIP transcription factor, as a target of KGB-1-mediated phosphorylation. We further identified two transcriptional targets of the KGB-1 pathway, *kreg-1* and *kreg-2*/*lys-3*, which are required for the defense against heavy metal stress. FOS-1 plays a critical role in the transcriptional repression of the *kreg-1* gene by recruiting histone deacetylase (HDAC) to its promoter. KGB-1 phosphorylation prevents FOS-1 dimerization and promoter binding, resulting in promoter derepression. Thus, HDAC behaves as a co-repressor modulating FOS-1-mediated transcriptional regulation. This study describes the direct link from JNK signaling, Fos phosphorylation, and regulation of *kreg* gene transcription, which modulates the stress response in *C. elegans*.

## Author Summary

We have investigated the mechanisms by which the soil nematode *Caenorhabditis elegans* regulates its response to environmental stresses. Previously, we showed that a conserved KGB-1 JNK mitogen-activated protein kinase (MAPK) pathway regulates the response to heavy metal stress in *C. elegans*, illustrating that these key stress signaling pathways in mammals are also conserved in *C. elegans*. Various proteins have been identified as potential targets of JNK MAPK in mammals; however, the identification of JNK MAPK physiological substrates has proven more challenging in *C. elegans*. Here we demonstrate that Fos, a bZIP family transcription factor, and HDAC, a Class I histone deacetylase, are crucial components functioning downstream of KGB-1 in the JNK-mediated stress response pathway. We find that FOS-1 functions as a transcriptional repressor by recruiting HDAC to its target promoters. In response to stress, activated KGB-1 relieves this repression via phosphorylation of FOS-1. Thus, this study shows how JNK signaling induces tolerance to stress at the gene level and describes a novel mechanism of gene regulation by which this is effected.

## Introduction

Mitogen-activated protein kinase (MAPK) signal transduction pathways are evolutionarily conserved in eukaryotic cells and transduce signals in response to a variety of extracellular stimuli. Each pathway is composed of three classes of protein kinases: MAPK, MAPK kinase (MAPKK) and MAPK kinase kinase (MAPKKK) [1], [2]. MAPKKK phosphorylates and activates MAPKK, which in turn activates MAPK. This activation cascade can be reversed by phosphatases. In particular, members of the MAPK phosphatase (MKP) family can remove phosphate groups from activated MAPK [1], [2]. Three subgroups of MAPKs have been identified: extracellular signal-regulated kinase (ERK), c-Jun N-terminal kinase (JNK), and p38 kinases [1], [2]. JNK and p38 MAPKs function as key mediators of stress and immune signaling in mammals. The MKK4 and MKK7 MAPKKs have been shown to activate JNK, and the MKK3 and MKK6 MAPKKs serve as the major activators of p38 MAPK. The specific MAPKKs are themselves phosphorylated and activated by specific MAPKKKs. Different MKPs display different activities toward ERK, JNK, and p38.

Invertebrate model organisms such as *Drosophila melanogaster* and *Caenorhabditis elegans* are useful for understanding the effects and interactions of JNK proteins, especially since they are amenable to the analysis of cytoprotective gene expression and the specific contributions of different tissues [3], [4]. Recent studies in *C. elegans* have revealed that the JNK MAPK signaling components are highly conserved between *C. elegans* and mammals. One such *C. elegans* JNK pathway is the KGB-1 pathway, composed of an MLK-type MAPKKK MLK-1, an MKK7-type MAPKK MEK-1 and a JNK-type MAPK KGB-1 [5]. The KGB-1 pathway is required for the protection against heavy metals and protein folding stress [5], [6], [7], and regulates the transcriptional responses to bacterial pore-forming toxins [8]. Another component of this pathway is the MKP VHP-1, which negatively regulates the KGB-1 pathway by dephosphorylating KGB-1 [5]. However, the components that function downstream of the KGB-1 pathway have yet to be elucidated.

Various targets of JNK phosphorylation have been identified in mammalian systems, including members of the basic region leucine zipper (bZIP) family of transcription factors such as ATF2 and Jun [9], [10]. The activating protein 1 complex (AP-1) constitutes an important subset of bZIP transcription factors [9], [10]. AP-1 component proteins interact as homodimers or heterodimers, bind DNA through conserved bZIP domains, and regulate transcription of their target genes. A large body of research supports a model in which extracellular stimuli trigger AP-1 phosphorylation by JNK, leading to reprogramming of target gene expression [11], [12]. Given the importance of chromatin dynamics in the control of gene expression, recent work has focused on factors interacting with AP-1 that can mediate chromatin modification and remodeling, notably enzymes that reversibly modify histone tails by acetylation. The histone deacetylase (HDAC) complex was thus found to inhibit the JNK pathway [13], [14]. Gene repression by the HDAC complex is relieved by phosphorylation of Jun, which causes it to dissociate from the promoter [15], [16]. These findings suggest that chromatin dynamics may play a central role in the cellular response to JNK signaling.

To understand the role of KGB-1 signaling in the heavy metal stress response, we screened for proteins that may interact with KGB-1 and identified FOS-1, a *C. elegans* homolog of Fos, and showed that it functions downstream of KGB-1. In addition, we identified two genes whose expression is induced by copper in a KGB-1-dependent manner: *kreg-1* and *kreg-2* (*KGB-1 regulated genes*). We found that FOS-1 represses transcription via the recruitment of a Class I histone deacetylase HDA-1 to the promoter. Biochemical assays demonstrated that phosphorylation by KGB-1 inhibits FOS-1 self-association and binding to the *kreg-1* promoter. These results suggest that FOS-1 and HDA-1 play an inhibitory role in the response to heavy metal stress, and that the KGB-1 pathway confers tolerance to heavy metals by phosphorylating and thereby negatively regulating FOS-1.

## Results

### KGB-1 interacts with and phosphorylates FOS-1

**Figure 1:**
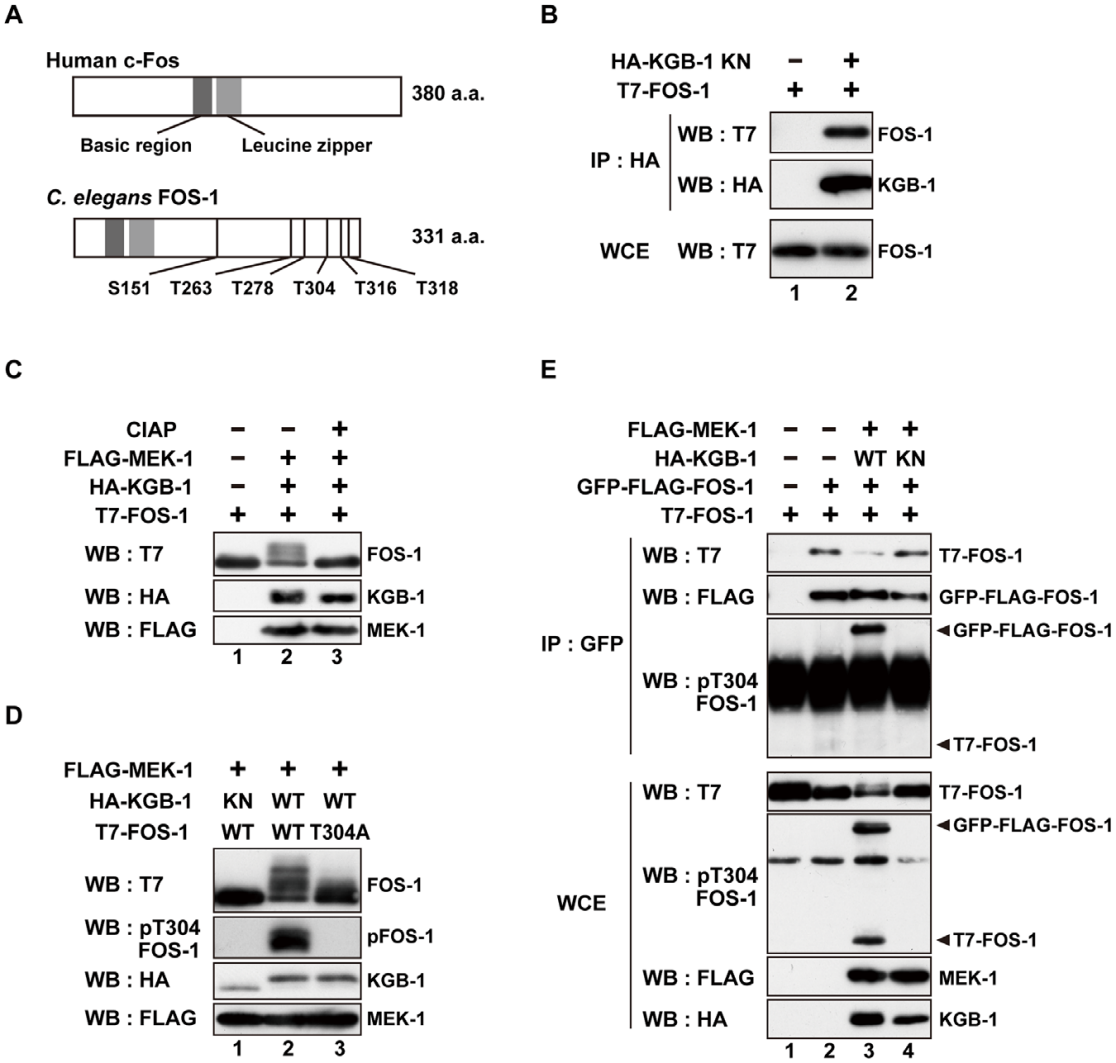
FOS-1 is phosphorylated by KGB-1. (A) Schematic representation of the structures of human c-Fos and *C. elegans* FOS-1 proteins. Dark boxes represent the basic and leucine zipper regions. Six Ser/Thr-Pro motifs are shown. (B) Interaction of FOS-1 with KGB-1. COS-7 cells were co-transfected with expression vectors encoding T7-FOS-1 and HA-KGB-1(K67R; KN) as indicated. Whole cell extracts (WCE) and immunoprecipitated complexes obtained with anti-HA antibodies (IP) were analyzed by Western blot (WB). Experiments were performed five times with similar results. (C, D) Phosphorylation of FOS-1 by KGB-1. COS-7 cells were co-transfected with expression vectors encoding T7-FOS-1 (wild type; WT), T7-FOS-1(T304A), HA-KGB-1 WT, HA-KGB-1 KN, and FLAG-MEK-1 as indicated. Whole cell extracts were incubated in either the absence or presence of calf intestine alkali phosphatase (CIAP) before analyzing by Western blot (C). Experiments were performed three times with similar results. (E) FOS-1 dimerization is inhibited by KGB-1-mediated phosphorylation. COS-7 cells were co-transfected with expression vectors encoding T7-FOS-1 WT, GFP-FLAG-FOS-1 WT, HA-KGB-1 WT, HA-KGB-1 KN, and FLAG-MEK-1 as indicated. Whole cell extracts and immunoprecipitated complexes obtained with anti-GFP antibodies were analyzed by Western blot (WB). Arrowheads indicate the positions of phosphorylated GFP-FLAG-FOS-1 and T7-FOS-1. Experiments were performed three times with similar results.

To identify components that function downstream of KGB-1, we screened a *C. elegans* mixed-stage cDNA library by the yeast two-hybrid method to isolate proteins that interact with KGB-1. Generally, kinase-negative (KN) forms of protein kinases constitutively associate with their substrate. Therefore, as bait we used KGB-1(K67R), a KN form in which Lys-67 in the ATP-binding motif has been mutated to arginine. From this screen, we identified 10 proteins that interact with KGB-1 (Table S1). One of them is FOS-1, an ortholog of the mammalian Fos transcription factor [10], [17]. Because Fos is a known substrate of MAPK in many systems, we considered FOS-1 as a likely substrate of KGB-1. The FOS-1 protein is similar to other Fos proteins in that it possesses a basic DNA-binding domain, a leucine zipper region, and a carboxyl terminus rich in serine and threonine residues, which are typical sites of phosphorylation (Figure 1A). The *fos-1* gene encodes two FOS-1 isoforms, FOS-1A and FOS-1B [17]. As FOS-1A has previously been characterized as a regulator of anchor-cell invasion during nematode development [17], we focused our investigations on the FOS-1B form (hereafter referred to as FOS-1). To confirm an interaction between KGB-1 and FOS-1, we co-expressed HA-tagged KGB-1 KN and T7-tagged FOS-1 in COS-7 cells, immunoprecipitated HA-KGB-1 KN with anti-HA antibodies, and probed for T7-FOS-1 on a Western blot with anti-T7 antibodies. We found that KGB-1 KN co-immunoprecipitated with FOS-1 (Figure 1B), indicating that KGB-1 can physically associate with FOS-1.

The physical association of KGB-1 with FOS-1 suggested that FOS-1 may be a phosphorylation target of KGB-1. Indeed, in COS-7 cells, co-expression of KGB-1 activated by MEK-1 resulted in the appearance of slower migrating forms of the FOS-1 protein when analyzed by SDS-polyacrylamide gel electrophoresis (SDS-PAGE) (Figure 1C, lane 2). Pre-treatment of extracts with alkali phosphatase reduced the intensities of the band shifts (Figure 1C, lane 3), which is a typical indicator of dephosphorylation. Expression of MEK-1 in the absence of KGB-1 did not induce any mobility shift (Figure S1A).

The FOS-1 protein contains six putative MAPK phosphorylation sites (S/TP): Ser-151, Thr-263, Thr-278, Thr-304, Thr-316, and Thr-318 (Figure 1A). We generated a mutant form of FOS-1, [FOS-1(6A)], in which all 6 Ser/Thr residues had been changed to Ala. When we analyzed extracts from COS-7 cells transfected with FOS-1(6A) together with active KGB-1, we observed no slowly migrating bands in SDS-PAGE (Figure S2A, lane 12). To identify the specific phosphorylated residue(s) in FOS-1, we introduced various combinations of Ala mutations into the six Ser/Thr residues. We observed that the T304A, T316A, T318A triple mutation completely abrogated phosphorylation of FOS-1 (Figure S2B, lane 9), suggesting that Thr-304, Thr-316, and/or Thr-318 are potential phosphorylation sites. We further generated three FOS-1 mutants that individually changed Thr-304, Thr-316, or Thr-318 to Ala and found that the FOS-1(T304A) mutation exhibited decreased phosphorylation by KGB-1 (Figure 1D, line 3 and Figure S2). These results suggest that T304 is a major site of phosphorylation. However, we did also observe a minor slower-migrating band, indicating that there is some residual phosphorylation of FOS-1(T304A) and that Thr-316 and/or Thr-318 may be minor sites of KGB-1 phosphorylation. To confirm that KGB-1 phosphorylates FOS-1 at the Thr-304 residue, we generated anti-phospho-FOS-1 antibodies that specifically recognize FOS-1 phosphorylated at Thr-304. Transfection with active KGB-1, but not with the kinase-negative mutant KGB-1 KN, resulted in strong reactivity of FOS-1 with this antibody (Figure 1D, lanes 1, 2). In contrast, we found that the FOS-1 (T304A) mutated form could not be detected by this antibody (Figure 1D, lane 3), confirming that it was specific for FOS-1 phosphorylated at Thr-304.

Fos family proteins function as dimers that bind DNA and regulate the transcription of target genes [9], [10], [18]. We therefore next investigated whether FOS-1 undergoes homo-dimerization. FOS-1 was fused to both GFP and FLAG and expressed in COS-7 cells together with T7-FOS-1. We immunoprecipitated the GFP-FLAG-FOS-1 protein with anti-GFP antibodies, and tested for co-precipitation of T7-FOS-1 by blotting with anti-T7 antibodies. We differentiated between GFP-FLAG-FOS-1 and T7-FOS-1 by virtue of their different molecular weights. Indeed, GFP-FLAG-FOS-1 readily co-immunoprecipitated with T7-FOS-1 (Figure 1E, lanes 1, 2), indicating that the two proteins oligomerized, presumably as dimers. We next examined whether KGB-1 phosphorylation correlated with the degree of FOS-1 self-association. Co-expression of active but not inactive KGB-1 resulted in reduced co-immunoprecipitation of T7-FOS-1 with GFP-FLAG-FOS-1 (Figure 1E, lanes 3, 4). We next examined the phosphorylation state of FOS-1 self-association using anti-phospho-FOS-1 antibodies and observed that the phosphorylated form of T7-FOS-1 was not co-precipitated with GFP-FLAG-FOS-1 (Figure 1E, lane 3). This indicates that phosphorylation inhibits self-association of FOS-1. We also generated a mutant intended to mimic FOS-1 phosphorylation by replacing the Thr-304 residue with glutamic acid, with the purpose to examine its self-association potential. However, when expressed in COS-7 cells, FOS-1(T304E) exhibited faster migration on SDS-PAGE compared to wild type FOS-1 (Figure S1B), suggesting that the structure of FOS-1(T304E) is different from that of phosphorylated FOS-1. Thus, this mutation does not appear to mimic FOS-1 phosphorylation.

### FOS-1 negatively regulates the stress response mediated by the KGB-1 pathway

**Figure 2:**
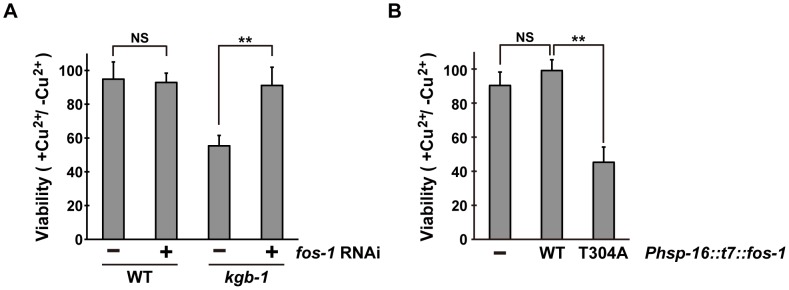
Effect of *fos-1* inhibition on stress sensitivity. (A) Suppression of the *kgb-1* heavy metal-sensitive phenotype by *fos-1* depletion. Each animal was cultured from embryogenesis on normal plates containing copper sulfate (40 µM) and seeded with a bacteria strain expressing the double-stranded RNA for *fos-1*. The relative viability is shown with standard errors. Error bars indicate 95% confidence interval. **P<0.01 as determined by Student's *t* test. NS, not significant. (B) Heavy metal sensitivity caused by FOS-1(T304A) overexpression. Wild-type animals harboring the *Phsp-16::t7::fos-1* transgene as an extrachromosomal array were cultured from embryogenesis on normal plates containing copper sulfate (100 µM). The relative viability is shown with standard errors. **P<0.01 as determined by Student's *t* test. NS, not significant.

Since the KGB-1 MAPK pathway regulates the response to heavy metal stress [5], [6], [7], we tested whether FOS-1 also regulates the stress response to heavy metals. Existing *fos-1* loss-of-function mutants could not be used to assay for heavy metal toxicity, because they have a sterile phenotype (data not shown). We therefore tested the effect of *fos-1* knockdown on the stress response using a feeding RNA interference (RNAi) method. Animals were placed on agar plates containing copper (Cu^2+^) ions, fed a bacteria strain expressing the double-stranded RNA for *fos-1*, and their development was monitored for any signs of an altered response to heavy metal stress. As shown Figure 2A, *fos-1* RNAi had no effect on the sensitivity to Cu^2+^ ions. Animals treated with *fos-1* RNAi exhibited an everted/protruded vulval phenotype in the adult as observed in *fos-1a* loss-of-function mutants [17]. This indicates that *fos-1* RNAi indeed had caused knockdown of *fos-1*. In contrast to the lack of effect in wild-type animals, *fos-1* RNAi suppressed the sensitivity to Cu^2+^ ions in *kgb-1(km21)* mutants (Figure 2A and Figure S3), suggesting that FOS-1 negatively regulates the tolerance to heavy metal stress.

The above results raised the possibility that KGB-1-mediated phosphorylation of FOS-1 Thr-304 relieves FOS-1-mediated inhibition in response to stress. To test this possibility, we expressed wild-type FOS-1 or the non-phosphorylatable FOS-1(T304A) mutant from the heat shock promoter (*Phsp-16*) in wild-type animals. We found that expression of FOS-1(T304A) resulted in sensitivity to Cu^2+^ ion compared to expression of wild type FOS-1 (Figure 2B). These results suggest that KGB-1 phosphorylation at Thr-304 negatively regulates FOS-1 function.

### Identification of genes whose transcription is activated by the KGB-1 pathway

**Figure 3:**
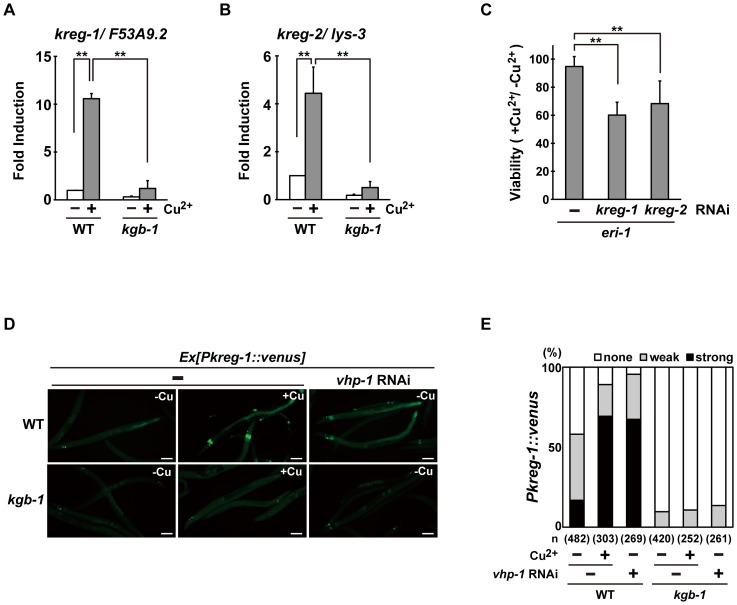
The KGB-1 pathway regulates expression of *kreg* genes. (A, B) Effect of copper ion on expression of *kreg-1* (A) and *kreg-2* (B). Wild-type and *kgb-1* mutant animals were cultured on plates seeded with a bacteria strain. At 3 days after hatching, animals were treated with copper sulfate (1 mM) for 1 hour and total RNA was isolated. Expression of genes was analyzed by qRT-PCR. Data are compared using a one-way ANOVA. **P<0.01. (C) Heavy metal sensitivity caused by inhibition of *kreg* genes. The *eri-1* mutant animals were cultured from embryogenesis on normal plates containing copper sulfate (100 µM) and seeded with bacteria strains expressing the indicated double-stranded RNA. The relative viability is shown with standard errors. Error bars indicate 95% confidence interval. **P<0.01 as determined by Student's *t* test. (D, E) Effect of copper ion on expression of the *kreg-1* reporter. Wild-type and *kgb-1* mutant animals harboring the *Pkreg-1::venus* transgene as an extrachromosomal array were cultured on plates seeded with a bacteria strain expressing the double-stranded RNA for *vhp-1*. At 3 days after hatching, animals were treated with copper sulfate (1 mM) for 1 hour. These animals were then transferred to NGM plates and incubated for 3 hours. Fluorescent (Venus) views are shown in D. Scale bar: 100 µm. “Weak” refers to animals in which intestinal Venus was present at low levels. “Strong” indicates that Venus was present at high levels in most of the intestine. Percentages of animals in each expression category are listed in E. The numbers (n) of animals examined are shown.

To understand how the KGB-1 pathway modulates gene activity and to define the physiological processes in which the heavy metal stress response may be involved, we examined gene expression changes in wild-type and *kgb-1* mutant animals subjected to heavy metal stress by carrying out a microarray analysis (see Materials and Methods) (Figure S4A and Tables S2, S3, S4, S5, S6, S7). From this, we identified six *kreg* (*KGB-1-regulated gene*) genes whose expression was regulated by KGB-1 (Figure S4B and Table S8). Among these, expression of two of the genes was increased in response to Cu^2+^ ions (Figure S4B and Table S8). These were designated *kreg-1* and *kreg-2*. The protein encoded by *kreg-1* (*F53A9.2*) is a novel 83 amino acids protein with polyhistidine streches, while the *kreg-2* gene is identical to *lys-3*, which encodes a lysozyme. We validated our microarray data by quantitative real-time RT-PCR (qRT-PCR) (Figure 3A and 3B). In wild-type animals, Cu^2+^ induced the expression of both *kreg-1* and *kreg-2*, but in *kgb-1(km21)* mutants induction of both genes was considerably reduced. To determine whether the *kreg* genes play functionally important roles in the resistance to heavy metal stress in *C. elegans* in vivo, we used RNAi to inhibit the expression of *kreg-1* or *kreg-2* and then examined the stress response. RNAis against either *kreg-1* or *kreg-2* caused a partial sensitivity to Cu^2+^ ions (Figure 3C and Figure S5). The *kreg-2*/*lys-3* gene encodes a secreted lysozyme that is presumably involved in anti-bacterial defense [19]. This raised the possibility that there may be a role for bacteria in the susceptibility to heavy metal stress. To test this possibility, we fed the worms on viable versus heat-killed bacteria and asked if this affected their heavy metal sensitivity. We found that heat treatment of bacteria had no effect on either the heavy metal sensitivity in wild-type animals or the heavy metal sensitive phenotype caused by *kgb-1* and *lys-3* mutations (data not shown). Thus, bacteria appear to play no role in the susceptibility to heavy metal stress and it remains unclear how LYS-3 may protect against heavy metal stress.

To analyze in vivo *kreg-1* expression patterns and to develop tools for further analysis, we generated a *Pkreg-1*::*venus* reporter, consisting of the *kreg-1* promoter driving expression of *venus*. Wild-type animals harboring the *Pkreg-1*::*venus* reporter exhibited weak Venus expression in the absence of Cu^2+^ (Figure 3D and 3E). However, *Pkreg-1::venus* expression was robustly induced in the intestine of animals following incubation with Cu^2+^ (Figure 3D and 3E). To confirm that the *Pkreg-1::venus* reporter behaves similarly to endogenous *kreg-1* mRNA, we tested whether *Pkreg-1::venus* induction is dependent on the KGB-1 MAPK pathway, which is negatively regulated by the VHP-1 phosphatase [5]. In contrast to the wild-type animals, very little *Pkreg-1::venus* expression was induced by Cu^2+^ in *kgb-1(km21)* mutants (Figure 3D and 3E). Treatment of animals with *vhp-1* RNAi resulted in the constitutive expression of the *Pkreg-1::venus* transgene in wild-type, but not in *kgb-1(km21)* animals (Figure 3D and 3E). Thus, the *Pkreg-1::venus* reporter is induced in response to heavy metal stress through the activation of the KGB-1 pathway.

### FOS-1 functions as a repressor of *kreg-1* induction mediated by the KGB-1 pathway

**Figure 4:**
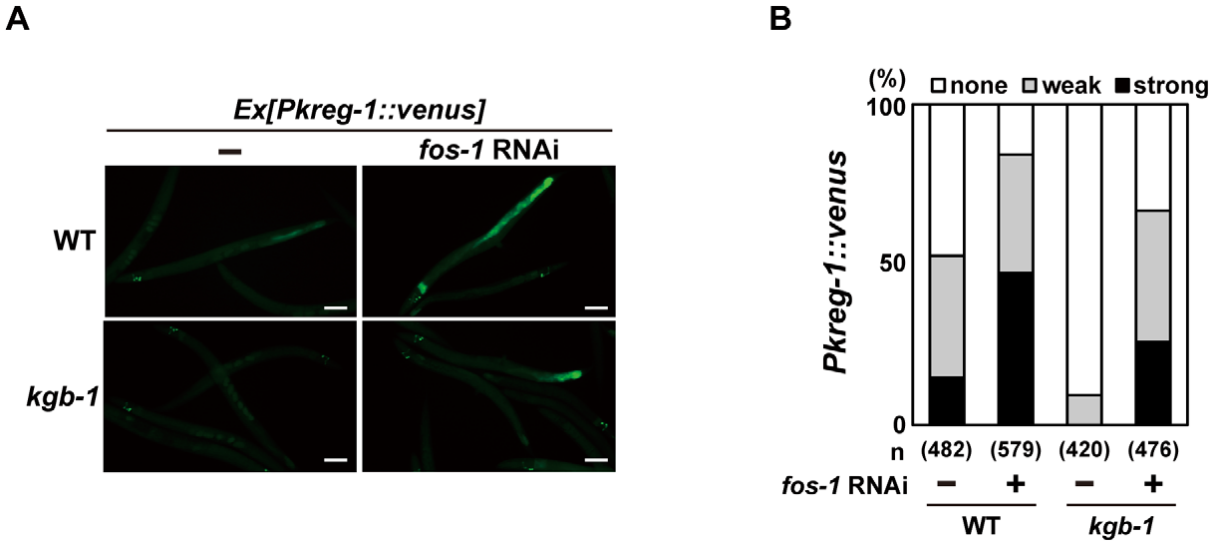
FOS-1 represses *kreg-1* expression. Wild-type and *kgb-1* mutant animals harboring the *Pkreg-1::venus* transgene as an extrachromosomal array were cultured on plates seeded with a bacteria strain expressing the double-stranded RNA for *fos-1*. Fluorescent (Venus) views are shown in A. Scale bar: 100 µm. “Weak” refers to animals in which intestinal Venus was present at low levels. “Strong” indicates that Venus was present at high levels in most of the intestine. Percentages of animals in each expression category are listed in B. The numbers (n) of animals examined are shown.

To understand the role of FOS-1 in the induction of *kreg-1* in response to Cu^2+^ stress, we examined the effect of *fos-1* RNAi on *Pkreg-1::venus* expression in *C. elegans*. Treatment with *fos-1* RNAi markedly increased intestinal *Pkreg-1*::*venus* expression even in the absence of Cu^2+^ (Figure 4). The effect of *fos-1* RNAi on expression of *kreg-1* and *kreg-2* was further confirmed by qRT-PCR (Figure S6). These results raised the possibility that FOS-1 functions as a repressor for gene induction activated by the KGB-1 pathway. To test this hypothesis, we carried out epistasis analysis using *fos-1* RNAi and *kgb-1(km21)* mutants. We observed that while expression of the *Pkreg-1::venus* reporter gene was diminished in *kgb-1(km21)* mutants, treatment with *fos-1* RNAi was epistatic to this and resulted in increased *kreg-1* reporter activity (Figure 4). This indicates that FOS-1 functions downstream of KGB-1 as a repressor of *kreg-1* induction by Cu^2+^.

Incubation with Cu^2+^ induced *Pkreg-1::venus* expression in the intestine in a manner dependent on the KGB-1 pathway. This observation suggests that activation of the KGB-1 pathway in the intestine is critical in the defense against heavy metal stress. Consistent with this, MEK-1, a MAPKK in the KGB-1 pathway, is expressed in intestinal cells [6], [20]. However, we have previously shown that expression of MEK-1 in the epidermis can rescue the Cu^2+^- sensitive phenotype of *mek-1* null mutants [6]. To test whether expression of MEK-1 in the intestine of *mek-1* mutants confers resistance to heavy metal stress, we expressed the *mek-1* cDNA in the intestine using the *elt-2* promoter. The *mek-1(ks54)* deletion mutant carrying *Pelt-2::mek-1* exhibited resistance to heavy metal stress (Figure S7). The *Pkreg-1::venus* reporter may lack the region required for its expression in epidermis.

Fos proteins bind to Jun or other bZIP proteins to create an AP-1 dimer complex, which regulates gene expression [9], [10], [18]. In fact, similar to mammalian and *Drosophila* Fos and Jun proteins, *C. elegans* FOS-1 and JUN-1 form heterodimers [18], [21]. To examine whether *C. elegans jun-1* plays the same role as *fos-1* in modulating *kreg-1* expression, we treated wild-type animals with *jun-1* RNAi, however it failed to increase intestinal *Pkreg-1*::*venus* expression (Figure S8A). ATF-7 is a member of the bZIP transcription factor family and functions in innate immunity mediated by the PMK-1 p38 pathway [22]. We therefore tested the effect of *atf-7* RNAi on *Pkreg-1::venus* reporter activity and similarly observed no effect (Figure S8A). Consistent with these results, neither knockdown of *jun-1* nor a loss-of-function *atf-7(qd22)* mutation resulted in enhanced heavy metal stress sensitivity in wild-type animals or suppression of the stress-sensitive phenotype of *kgb-1* mutants (Figure S8B and S8C). Thus, JUN-1 and ATF-7 do not participate in the heavy metal stress response mediated by the KGB-1 pathway.

**Figure 5:**
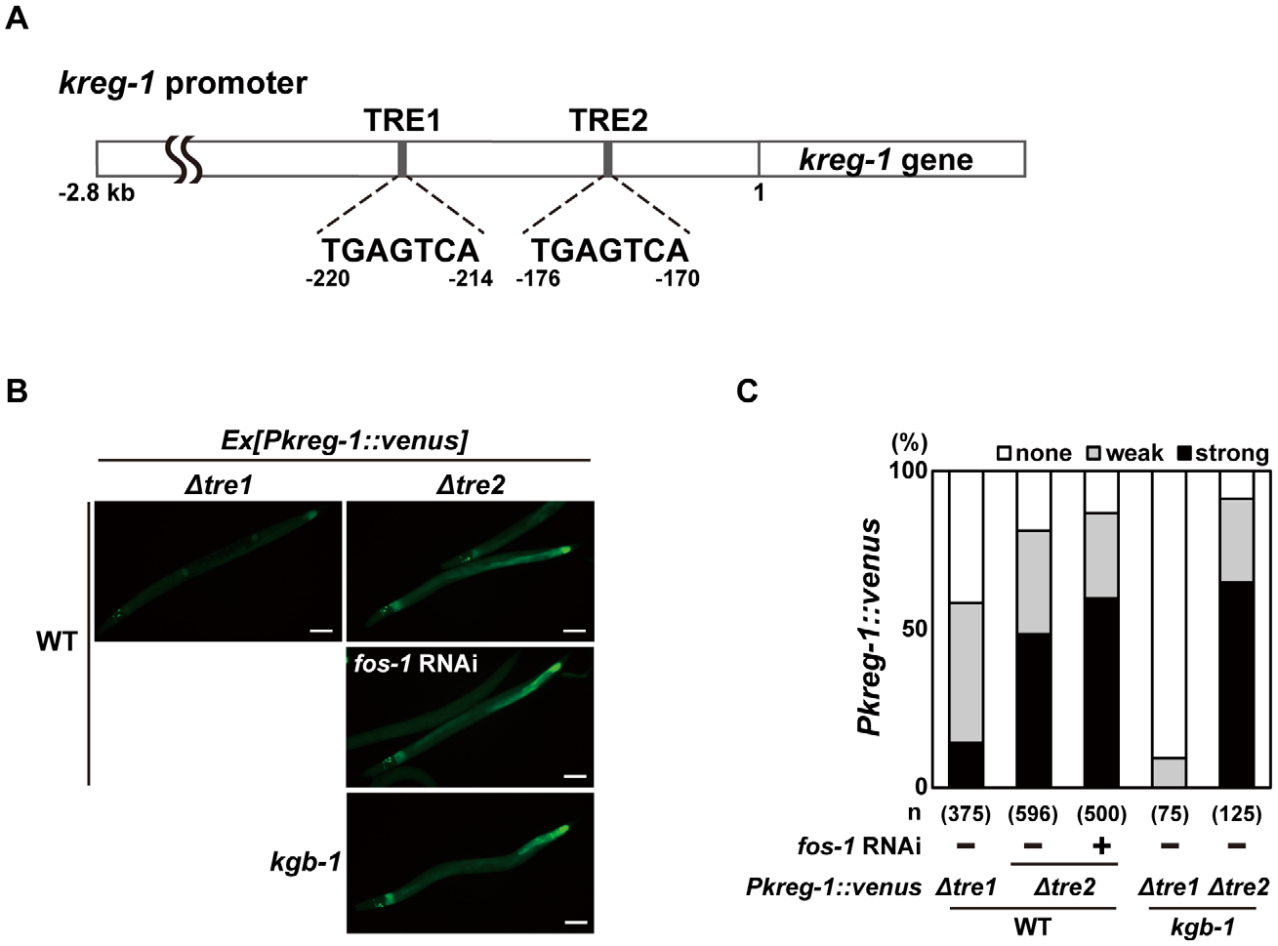
FOS-1 negatively regulates *kreg-1* expression via the TRE2 site. (A) Schematic representation of the structure of the *kreg-1* promoter. Two TRE sites are represented by dark boxes. (B, C) Effect of deletion of the TRE sites on expression of the *kreg-1* reporter. Wild-type and *kgb-1* mutant animals harboring the *Pkreg-1Δtre1::venus* or *Pkreg-1Δtre2::venus* transgene as an extrachromosomal array were cultured on plates seeded with a bacteria strain expressing the double-stranded RNA for *fos-1*. Fluorescent (Venus) views are shown in B. Scale bar: 100 µm. “Weak” refers to animals in which intestinal Venus was present at low levels. “Strong” indicates that Venus was present at high levels in most of the intestine. Percentages of animals in each expression category are listed in C. The numbers (n) of animals examined are shown.

The bZIP domain of Fos binds the consensus sequence, TGA(C/G)TCA, called the TPA-responsive element (TRE) [23]. The promoter region of the *kreg-1* gene contains two TRE binding motifs, termed TRE1 and TRE2 (Figure 5A). To determine whether these TRE motifs are required for FOS-1-mediated repression of *Pkreg-1::venus* expression, we deleted each motif independently within the *Pkreg-1::venus* reporter (Figure 5A). Deletion of TRE1 (*Pkreg-1Δtre1::venus*) had no effect on the expression pattern of the transgene (Figure 5B and 5C). In contrast, deletion of TRE2 (*Pkreg-1Δtre2::venus*) resulted in constitutive expression in both wild-type and *kgb-1(km21)* mutant animals (Figure 5B and 5C). Furthermore, we found that treatment with *fos-1* RNAi did not enhance constitutive expression of the *Pkreg-1Δtre2::venus* transgene (Figure 5B and 5C). Thus, the TRE2 binding site is required in cis to mediate repression of *kreg-1* by FOS-1. These results support the possibility that FOS-1 negatively regulates *kreg-1* expression through the TRE2 site in the promoter.

**Figure 6:**
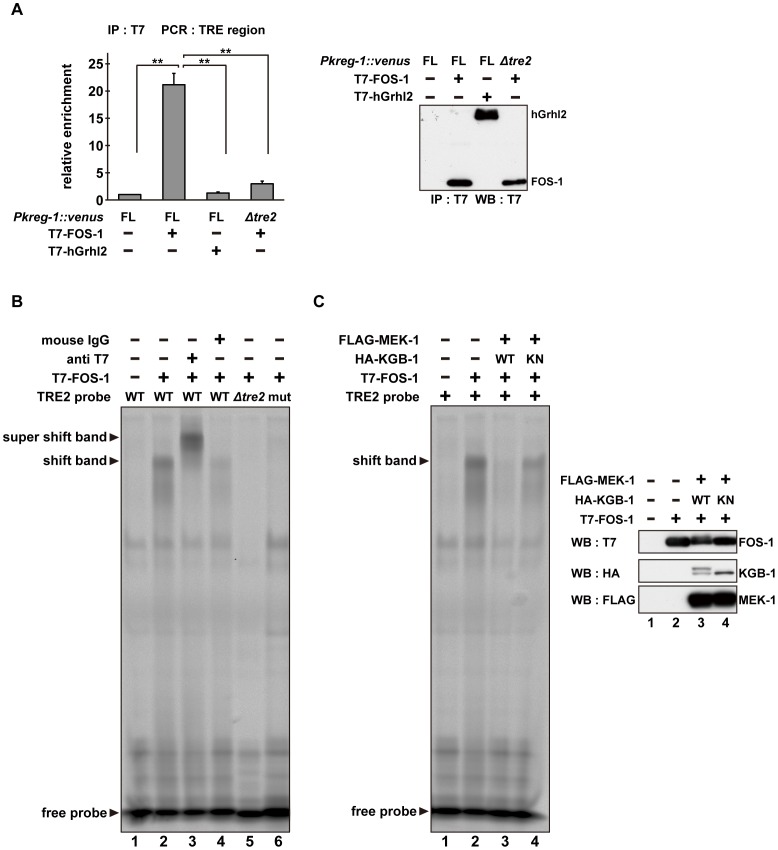
The DNA binding activity of FOS-1 is inhibited by KGB-1-mediated phosphorylation. (A) FOS-1 binds to the TRE2 sites. HEK293 cells were co-transfected with the *Pkreg-1::venus* construct together with expression vectors encoding T7-FOS-1 or T7-hGrhl2 as indicated. For chromatin immunoprecipitation assays, immunoprecipitated complexes obtained with anti-T7 antibodies were analyzed by quantitative PCR. Data are compared using a one-way ANOVA. **P<0.01. Immunoprecipitated T7-FOS-1 and T7-hGrhl2 were monitored by Western blot. (B, C) Effect of FOS-1 phosphorylation by KGB-1 on the TRE2 binding activity. COS-7 cells were co-transfected with expression vectors encoding T7-FOS-1, HA-KGB-1 WT, HA-KGB-1 KN, and FLAG-MEK-1 as indicated. For gel retardation assays, cell extracts were incubated with the TRE2 retardation probes. Anti-T7 antibodies or normal mouse IgG were added in the binding reactions (B). Expression of T7-FOS-1, HA-KGB-1, and FLAG-MEK-1 was monitored by Western blot. Experiments were performed three times with similar results.

To examine whether FOS-1 binds directly to the *kreg-1* promoter via TRE2, we conducted chromatin immunoprecipitation (ChIP) assays. Human embryonic kidney (HEK) 293 cells were co-transfected with the *Pkreg-1*::*venus* reporter together with either T7-FOS-1 or the negative control T7-hGrhl2. Lysates were immunoprecipitated with anti-T7 antibodies, and quantitative PCR analysis was performed to amplify DNA fragments contained in the immunoprecipitated complexes. PCR analysis showed that FOS-1 bound efficiently to the *kreg-1* promoter, whereas the negative control human Grhl2 protein did not (Figure 6A). We could detect binding of FOS-1 to the *Pkreg-1Δtre1::venus* transgene (data not shown), but not to the *Pkreg-1Δtre2::venus* transgene (Figure 6A), indicating that FOS-1 associates with the *kreg-1* promoter via an interaction with the TRE2 motif.

As shown above, self-association of FOS-1 is prevented by KGB-1-mediated phosphorylation. We next addressed whether FOS-1 phosphorylation affects its ability to interact with the TRE2 element of the *kreg-1* promoter. Cell extracts obtained from COS-7 cells expressing T7-FOS-1 were incubated with probes and analyzed in a gel-retardation assay. We found that FOS-1 was able to associate with a probe containing the optimal TRE2 sequence, but not with a probe in which the core 6 bases of TRE2 were deleted (Figure 6B, lanes 1, 2, 5). To further confirm the interaction of FOS-1 with the TRE2 element, we utilized site-directed mutagenesis to convert the consensus TGAGTCA sequence to AAGCTTA in the TRE2 element. A similar alteration has been shown to inhibit the AP-1-DNA interaction [24]. Indeed, we observed that FOS-1 was not able to bind to the mutated TRE2 probe (Figure 6B, lane 6). In addition, the protein-DNA complex was supershifted by pre-incubation with anti-T7 antibody (Figure 6B, lane 3), indicating that T7-FOS-1 is involved in this complex. When MEK-1 and KGB-1 were co-expressed with T7-FOS-1 in COS-7 cells, the association of FOS-1 with the optimal TRE2 probe was decreased (Figure 6C, lanes 1–3). This reduction was dependent on the kinase activity of KGB-1 (Figure 6C, lane 4). Thus, FOS-1 phosphorylation by KGB-1 decreases the association of FOS-1 with its target gene promoter. Taken together, these results suggest that the KGB-1 pathway activates transcription of target genes by phosphorylation of FOS-1, which inhibits FOS-1 self-association and binding to its target promoter.

### *C. elegans* histone deacetylase HDA-1 functions as a negative regulator of *kreg-1* induction mediated by the KGB-1 pathway

**Figure 7:**
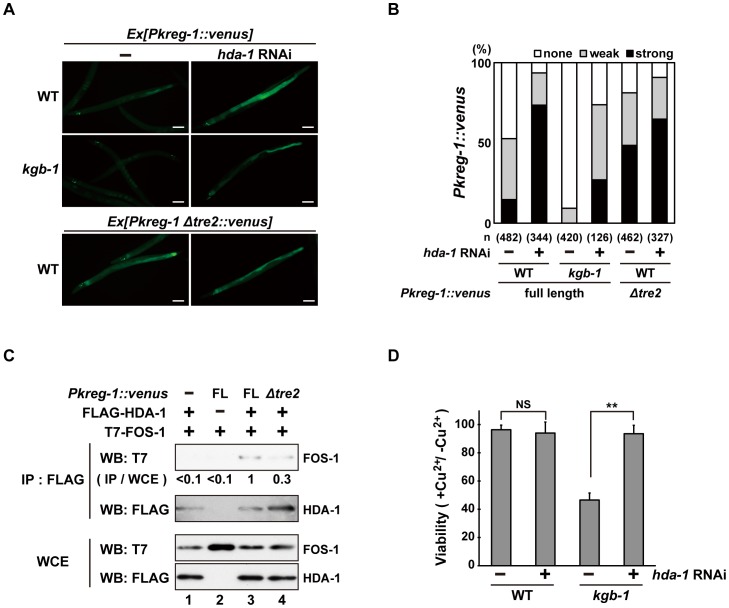
HDA-1 functions cooperatively with FOS-1. (A, B) Effect of *hda-1* depletion on expression of the *kreg-1* reporter. Wild-type and *kgb-1* mutant animals harboring the *Pkreg-1::venus* transgenes as an extrachromosomal array were cultured on plates seeded with a bacteria strain expressing the double-stranded RNA for *hda-1*. Fluorescent (Venus) views are shown in A. Scale bar: 100 µm. “Weak” refers to animals in which intestinal Venus was present at low levels. “Strong” indicates that Venus was present at high levels in most of the intestine. Percentages of animals in each expression category are listed in B. The numbers (n) of animals examined are shown. (C) Interaction of HDA-1 with FOS-1. HEK293 cells were co-transfected with the *Pkreg-1::venus* construct and expression vectors encoding FLAG-HDA-1 and T7-FOS-1 as indicated. Whole cell extracts and immunoprecipitated complexes obtained with anti-FLAG antibodies were analyzed by Western blot. FOS-1 signal intensities in co-immunoprecipitates with HDA-1 were quantitated and normalized to those in whole cell extracts. Relative levels of immunoprecipitated FOS-1 are shown. Experiments were performed three times with similar results. (D) Suppression of the *kgb-1* heavy metal-sensitive phenotype by *hda-1* depletion. Each animal was cultured from embryogenesis on normal plates containing copper sulfate (40 µM) and seeded with a bacteria strain expressing the double-stranded RNA for *hda-1*. The relative viability is shown with standard errors. Error bars indicate 95% confidence interval. **P<0.01 as determined by Student's *t* test. NS, not significant.

How does FOS-1 repress *kreg-1* transcription? Given the importance of chromatin dynamics in the control of gene expression, recent work has focused on AP-1 interaction partners capable of chromatin remodeling and modification [13]–[16], [25], [26]. It has been reported that AP-1, during the innate immune response, recruits HDAC1, a member of the Class I histone deacetylase (HDAC) family, to the promoter of a gene that encodes an antibacterial protein where it deacetylates promoter-associated histones [26]. Therefore, we examined whether HDACs might affect *Pkreg-1::venus* expression. *C. elegans* possesses three HDAC genes, *hda-1*, *hda-2* and *hda-3*, which encode Class I HDAC homologs [27], [28]. We found that treatment with *hda-1* RNAi resulted in constitutive expression of the *Pkreg-1::venus* reporter in wild-type animals (Figure 7A and 7B). Furthermore, *hda-1* knockdown significantly restored loss of intestinal *Pkreg-1*::*venus* expression in *kgb-1(km21)* mutants (Figure 7A and 7B). We also found that *hda-1* RNAi had little effect on the constitutive expression caused by the *Δtre2* deletion of the *Pkreg-1::venus* reporter (Figure 7A and 7B), indicating that negative regulation of *kreg-1* expression by HDA-1 requires the TRE2 motif in the promoter. In addition, we observed by qRT-PCR that *hda-1* RNAi enhanced expression of the *kreg-2* gene (Figure S9), confirming that this effect is not specific only to *kreg-1*.

Next we asked whether FOS-1 could interact with HDA-1. T7-FOS-1 and FLAG-HDA-1 were co-expressed in HEK293 cells. We immunoprecipitated FLAG-HDA-1 with anti-FLAG antibodies, and probed for the T7-FOS-1 on a Western blot with anti-T7 antibodies. We failed to detect an association between FOS1- and HDA-1 (Figure 7C, lane 1). However, if we transfected in the *Pkreg-1::venus* reporter along with T7-FOS-1 and FLAG-HDA-1, we could detect an association between FOS-1 and HDA-1 (Figure 7C, lane 3). Furthermore, removal of the TRE2 site from the *Pkreg-1*::*venus* reporter reduced this interaction (Figure 7C, lane 4). These results suggest that HDA-1 and FOS-1 can associate on the *kreg-1* promoter.

Finally, we examined whether HDA-1 contributes to the response to heavy metal stress. Knockdown of *hda-1* by RNAi in wild-type animals had no effect on their sensitivity to Cu^2+^ ions (Figure 7D). In contrast, knockdown of *hda-1* by RNAi suppressed the sensitivity to Cu^2+^ ions in *kgb-1(km21)* mutants. Thus, HDA-1 negatively regulates the heavy metal stress response, consistent with the observation that *kreg-1* expression is repressed by HDA-1.

## Discussion

**Figure 8:**
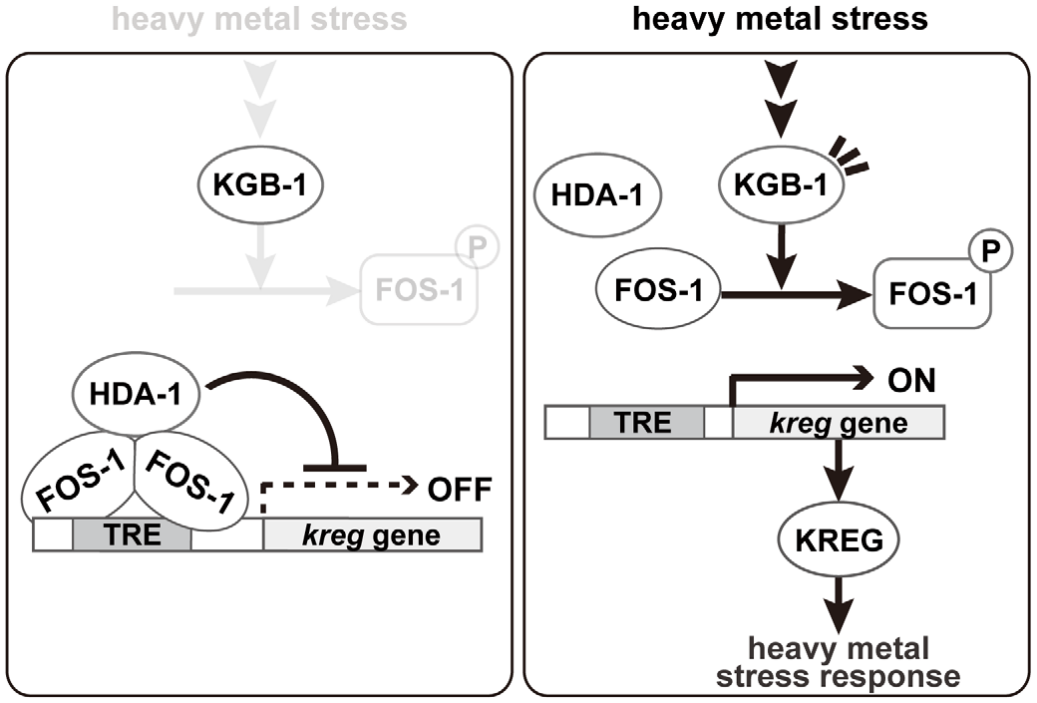
Proposed model for the KGB-1 pathway in stress response. In the absence of heavy metal stress, FOS-1 forms homodimers and binds to the TRE2 motif in the *kreg* target promoter. FOS-1 dimerization potentiates recruitment of HDA-1 to the promoter. The FOS-1/HDA-1 repressor complex represses transcription of *kreg* target genes (left panel). In the presence of heavy metal stress, the KGB-1 pathway is activated and FOS-1 is phosphorylated by KGB-1. FOS-1 phosphorylation leads to a switch from dimer to monomer, resulting in dissociation of the FOS-1/HDA-1 repressor complex from the target promoter that activates transcription of *kreg* genes (right panel).

JNK MAPK cascades are pivotal signaling modules controlling diverse signal transduction pathways in eukaryotes. The *C. elegans* KGB-1 JNK pathway regulates the stress response to heavy metals [5], [6], [7]. In this study, we present functional evidence showing that FOS-1, a bZIP transcription factor homologous to human Fos, and HDA-1, a member of the Class I histone deacetylase family, are crucial components functioning downstream in the KGB-1-mediated stress response pathway (Figure 8). In the absence of stress, FOS-1 and HDA-1 act cooperatively to repress transcription of target genes involved in the heavy metal stress response. In response to stress, activated KGB-1 relieves this repression by phosphorylating FOS-1. Thus, we provide a mechanistic linkage between FOS-1 phosphorylation, the degree of its dimerization and its biological activity/function.

### Identification of target genes downstream of the KGB-1 pathway

A key step in understanding the KGB-1 JNK pathway is the identification of downstream targets that are activated by KGB-1 and that perform the actual protective function. Analysis of gene expression comparing wild type and *kgb-1* mutants has led to the identification of two targets of the KGB-1 pathway, namely *kreg-1* and *kreg-2*/*lys-3*. Both targets are transcriptionally induced by stress, both require the KGB-1 pathway for their full induction, and both are required for protection of the animal against heavy metal stress. These data suggest that activation of the KGB-1 pathway leads to increased production of these proteins that this in turn leads to protection and defense against heavy metal stress.

The identity of one of these genes is particularly revealing: The protein encoded by *kreg-1* contains polyhistidine stretches, which are well known to bind metal ions (e.g. Ni^2+^, Cu^2+^, Co^2+^ and Zn^2+^) and widely used as an affinity tag [29]. A previous study also revealed that Hpn, a 60 amino acids protein with polyhistidine stretches in *Helicobacter pylori*, preferentially binds Cu^2+^ ion and is able to confer copper resistance when expressed in *Escherichia coli* [30]. Thus, we speculate that the KREG-1 protein may confer resistance to Cu^2+^ stress by chelating this ion through these polyhistidine stretches.

### Role of FOS-1 in the KGB-1 pathway

In this study, we identified the FOS-1 bZIP transcription factor as a downstream component of the KGB-1 pathway. FOS-1 was isolated as a protein that binds to KGB-1 and we showed that KGB-1 phosphorylates FOS-1 in the C-terminal regulatory region. Fos and Jun of bZIP transcription factors form part of the AP-1 transcription factor complexes [18], [23]. These transcription factors are homologous within two adjacent domains: a basic region and a leucine zipper motif, which are necessary for DNA binding and factor dimerization, respectively. Indeed, *C. elegans* FOS-1 acts as an activator of spermathecal-specific *plc-1* gene expression by forming heterodimers with JUN-1 [21]. In addition, a genome-wide RNAi screen identified *fos-1* and *jun-1* as genes important for the KGB-1-mediated defense pathway against pore-forming toxins made by soil bacterium [8]. Thus, it is likely that the JNK-AP-1 pathway has the role in protection against pore-forming toxins by regulating transcriptional responses. However, we found that JUN-1 is not involved in the KGB-1-mediated stress response pathway. We demonstrated that FOS-1 is capable of forming homodimers and acts as a repressor of its target gene expression. Dimerization of FOS-1 most likely serves to enhance its DNA binding affinity to target promoters and it is therefore likely that the *C. elegans* FOS-1 binding partner determines whether FOS-1 functions as a repressor or activator.

It has been proposed that bZIP transcription factors can switch between repressor and activator mode, as illustrated by the transcriptional regulation of *C. elegans* ATF-7 and the yeast Sko1p resulting from MAPK activation [22], [31]. Activation of the PMK-1 p38 MAPK pathway in response to pathogen infection results in PMK-1 phosphorylation of ATF-7, leading to a switch in ATF-7 from a transcriptional repressor to an activator [22]. In yeast, Sko1p is phosphorylated via the Hog1p MAPK pathway in response to osmotic stress, and this converts Sko1p from a repressor to an activator [31]. Here, we found that depletion of FOS-1 suppressed the heavy metal sensitivity of *kgb-1* mutants, but had no effect on the heavy metal sensitivity in wild-type animals. These results strongly suggest that FOS-1 simply acts as a transcriptional repressor of the heavy metal stress response mediated by the KGB-1 pathway. Thus, FOS-1 regulation of the heavy metal stress response does not appear to involve switching of its transcriptional regulation activity.

Our analysis showing FOS-1 phosphorylation by KGB-1 and its biological consequences has provided some novel molecular insights into the regulation of FOS-1. We found that phosphorylation blocks FOS-1 dimer formation and that this results in reduced binding to the promoter of target genes. We imagine that dimeric FOS-1 binds DNA with a higher affinity than the monomeric form. Based on these data, we propose that activation of the KGB-1 pathway in response to heavy metal stress results in FOS-1 phosphorylation, leading to a switch of FOS-1 from dimer to monomer and consequent loss of promoter binding (Figure 8).

### Mechanism of FOS-1/HDA-1-mediated control of gene expression in the KGB-1 pathway

How does FOS-1 act as a repressor of *kreg-1* transcription? Our results suggest that the HDA-1 histone deacetylase co-operates with FOS-1 to repress transcription of the *kreg-1* gene (Figure 8). Many transcription factors have been shown to recruit protein complexes that locally alter the acetylation of histones. Recruitment of HDAC can lead to transcriptional repression, whereas recruitment of histone acetyltransferase can lead to transcriptional activation. These results suggest that FOS-1 acts as a transcriptional repressor by recruiting HDA-1 to the promoter of the *kreg-1* gene. Therefore, it is quite likely that KGB-1 activates *kreg-1* expression by derepressing this FOS-1/HDA-1 repressor complex (Figure 8). In this model, FOS-1 forms homodimers and binds to the TRE2 motif in the *kreg-1* promoter. FOS-1 dimerization might also potentiate the recruitment of HDA-1 to the promoter. Thus, the FOS-1/HDA-1 repressor complex may function to prevent inadvertent activation of the *kreg* genes in the absence of heavy metal stress. When signaled by heavy metal stress, KGB-1 is activated and phosphorylates FOS-1, which leads to dissociation of the FOS-1 dimer and dissociation of the FOS-1/HDA-1 repressor complex from the *kreg-1* promoter, resulting in the activation of *kreg-1* expression.

The ability of Fos to function as a repressor has also been described in *Drosophila* [14]. HDAC is recruited to promoters occupied by unphosphorylated DFos and represses transcription of its target genes. JNK-mediated phosphorylation of DFos not only releases the HDAC corepressor complex and leads to activation by derepression but also unmasks the function of histone acetyltransferase and results in increased transcriptional efficiency. However, the mechanism of *C. elegans* FOS-1 derepression described here represents a unique case where transcription factor phosphorylation leads to reduced dimerization, DNA binding and loss of HDAC association. Comparing *Drosophila* Fos and *C. elegans* FOS-1, we find that significant homology is present only in the adjacent basic and leucine zipper motifs. In addition, the amino acid sequence of the region flanking the phosphorylation sites is not conserved between *Drosophila* Fos and *C. elegans* FOS-1 [32]. Nevertheless, the basic mechanisms of JNK-mediated phosphorylation of Fos and its effects on Fos/HDAC repressor complex formation are evolutionally conserved between *C. elegans* and *Drosophila*. This finding thus reveals a common underlying mechanism by which the JNK signaling pathway modulates the activities of the Fos family of bZIP transcription factors.

In summary, we have described a mechanism of transcriptional regulation whereby KGB-1 activates expression of the stress response genes by promoting the dissociation of a FOS-1/HDA-1 repressor complex. This is a new finding that could provide valuable insights into the stress response in the context of the whole organism. It would greatly enhance our understanding of the stress response mediated by JNK signaling to elucidate how the *kreg* genes confer tolerance to heavy metals in *C. elegans*.

## Materials and Methods

### Plasmids

The yeast expression vector for the LexA DNA-binding domain (DBD)-fused KGB-1(K67R) was constructed by inserting each coding sequence into pBTM116. The mammalian expression vectors for HA epitope-tagged KGB-1 (HA-KGB-1) and FLAG epitope-tagged MEK-1 (FLAG-MEK-1) were described previously [5]. The cDNA for *fos-1* was isolated by the Y. Kohara EST project (National Institute of Genetics, Mishima, Japan). The cDNAs for *hda-1* and human *Grhl2* were amplified by PCR from *C. elegans* and human cDNA libraries, respectively, and completely sequenced. The mammalian expression constructs for T7-FOS-1, GFP-FLAG-FOS-1, FLAG-HDA-1 and T7-hGrhl2 were constructed by inserting each coding sequence into a vector expressing epitope-tagged protein under the control of the cytomegalovirus (CMV) promoter. Each coding sequence was amplified by PCR using primer sets to create restriction sites immediately before the first codon and after the stop codon. Mutated forms of FOS-1 were made by oligonucleotide-directed PCR and the mutations were verified by DNA sequencing. To construct the *Phsp-16::t7::fos-1* plasmids, each *t7::fos-1* fragment from the mammalian expression vectors for T7-FOS-1 was subcloned into the pPD49.78 vector. Gateway cloning technology (Invitrogen) was used to construct the *Pkreg-1*::*venus* plasmid for expression in animals. The *Pkreg-1*::*venus* plasmid was constructed by fusion of the *venus* coding sequence to a 2.8 kbp genomic fragment containing the *kreg-1* promoter. Deletions of *Pkreg-1*::*venus* were made by oligonucleotide-directed PCR and the deletions were verified by DNA sequencing. The *Pelt-2::mek-1::venus* plasmid was constructed by fusing three DNA fragments in the following order: a 2.9 kbp genomic fragment containing the *elt-2* promoter, the *mek-1* coding sequence, and the *venus* coding sequence. The *Pmek-1::mek-1::venus*, *Pttx-3::gfp* and *sur-5::gfp* plasmids were described previously [6], [33], [34].

### Antibodies

Anti-phospho-FOS-1 rabbit polyclonal antibody was raised against a synthetic phospho-polypeptide, CSNTGL(P)TPSGQP [(p), phosphorylated], which corresponds to the C-terminal portion of FOS-1 and affinity purified. Anti-HA monoclonal antibody 16B12 (Covance), anti-FLAG monoclonal antibody M2 (Sigma), anti-T7 monoclonal antibody (Novagen) and anti-GFP polyclonal antibody (Clontech) were used.

### *C. elegans* strains

All strains were maintained on nematode growth medium (NGM) plates at 20°C and fed with bacteria of the OP50 strain, as described [35]. The alleles used in this study were N2 Bristol as the wild type, *kgb-1(km21)*, *mek-1(ks54)*, *atf-7(qd22)*, and *eri-1(mg366)*. Strains carrying the *Phsp-16::t7::fos-1* transgene were generated by injecting this DNA together with the *sur-5::gfp* plasmid, which expresses GFP in the nuclei of most somatic cells from embryogenesis, into the gonads of young adult N2 animals as described [36]. Strains carrying the *Pkreg-1*::*venus* transgene were generated by injecting this DNA together with the *Pttx-3::gfp* plasmid, which expresses GFP in a pair of AIY interneurons, into the gonads of young adult N2 animals.

### Stress sensitivity

Assays for the effect of *fos-1* transgenes on heavy metal toxicity were carried out as follows. Animals were grown and allowed to lay eggs on NGM plates seeded with bacteria of the OP50 strain. Embryos expressing GFP were transferred to NGM plates containing the indicated concentrations of copper sulfate. After incubation for 1 day at 20°C, the numbers of hatched embryos were determined by counting unhatched embryos. After additional incubation for 3 days either at 20°C or 33°C for 1 hour twice a day, the animals that developed into adulthood were counted. The percentage of adults was calculated by multiplying the number of adults by 100 and dividing by the number of hatched animals. The relative viability was estimated by dividing the percentage of adults in the presence of heavy metals by the percentage of adults in the absence of heavy metals.

Assays for the effect of RNAi on heavy metal toxicity were performed as follows. Animals were grown and allowed to lay eggs on NGM plates seeded with bacteria of the OP50 strain. Embryos were transferred to NGM plates containing the indicated concentrations of copper sulfate and seeded with bacteria of the HT115 strain carrying plasmids expressing the respective double-stranded RNAs for *fos-1*, *kreg-1*, *kreg-2*, *jun-1* or *hda-1*. After incubation for 1 day at 20°C, the numbers of hatched embryos were determined by counting unhatched embryos. The animals that developed into adulthood were counted 4 days after egg laying. The relative viability was estimated as described above.

### RNA isolation, microarray, and real-time qRT–PCR

Adult worms of each strain were incubated with H_2_O or 1 mM copper sulfate for 1 hour. Total RNA was then prepared using Trizol reagent (Invitrogen), followed by DNase I treatment, phenol/chloroform extraction and ethanol precipitation. RNA was dissolved in water and used as a template for a genome-wide microarray analysis and real-time qRT-PCR. Affymetrix GeneChip microarray processing was performed once by Takara Bio Inc. according to the manufacturer's protocol (Affymetrix). Briefly, total RNA was prepared from wild-type and *kgb-1* mutant animals subjected to Cu^2+^ ion exposure or left untreated (control). Biotinylated cRNA was hybridized to Affymetrix Genechips containing probes against 22,500 transcripts. qRT-PCR was performed with a 7300 real-time RT-PCR system (Applied Biosystems) using SYBR Premix Ex Taq (Takara). A standard curve was generated from diluted RNA derived from wild-type animals, and levels of gene expression were normalized to *act-1* expression.

### Identification of *kreg* genes

The microarray results were used as an initial screen to identify genes whose expression was increased in response to Cu^2+^ ions and in a manner dependent on KGB-1. We selected target genes by the following process (Figure S4A). First, transcript expression levels were compared between animals with or without Cu^2+^ treatment (Tables S2 and S3). 334 genes were chosen that were up-regulated greater than 2-fold by Cu^2+^ in wild-type animals (Table S4). Second, we compared Cu^2+^-mediated gene induction in wild-type versus *kgb-1* mutant animals to identify genes whose induction was affected by *kgb-1*. We identified 66 genes whose induction by Cu^2+^ in *kgb-1* mutants was <50% of the induction seen in wild-type animals (Table S5). Third, we compared basal expression levels between wild-type and *kgb-1* mutant animals, since basal activity of KGB-1 can be detected in wild-type animals [5], [6]. We identified 50 genes whose basal expression was decreased or not changed in *kgb-1* mutants versus wild-type animals (Table S6). Finally, data were manually curated to remove genes no longer predicted to be expressed using data available in Wormbase. From this we chose the top 13 genes whose expression was significantly induced by Cu^2+^ in wild-type animals (Table S7). We then re-examined regulation of these genes in a more quantitative manner by qRT-PCR (Figure S4B). From this we obtained a final list of 6 genes whose regulation was reproducibly affected by *kgb-1* (Table S8).

Microarray data for the Cu^2+^-treated/non-treated wild-type animals and Cu^2+^-treated/non-treated *kgb-1* mutant animals have been deposited in NCBI-GEO with the accession numbers GSE42703. The following links have been created to allow review of records GSE42703: http://www.ncbi.nlm.nih.gov/geo/query/acc.cgi?acc=GSE42703

### Reporter assay

Wild-type and *kgb-1* mutant animals harboring the *Pkreg-1::venus* transgene as an extrachromosomal array were cultured on plates seeded with a bacteria strain expressing the respective double-stranded RNAs for *vhp-1*, *fos-1*, *jun-1*, *atf-7 or hda-1*. At 3 days after hatching, animals were treated with copper sulfate (1 mM) for 1 hour. These animals were then transferred to NGM plates and incubated for 3 hours. The percentages of animals in each expression category are listed. “Weak” refers to animals in which intestinal Venus was present at low levels. “Strong” indicates that Venus was present at high levels in most of the intestine.

### Phosphatase treatment, immunoprecipitation, and ChIP assays

For phosphatase treatment, cell lysates were incubated with or without calf intestinal alkaline phosphatase (NEB) at 36°C for 5 minutes. Immunoprecipitation from COS-7 cells was carried out as described previously [37]. For immunoprecipitation from HEK293 cells, cells were pretreated with 1% paraformaldehyde in PBS for 10 minutes and glycine at a final concentration of 0.125 M for 5 minutes and collected. The ChIP assay was performed using ChIP-IT Express Enzymatic Shearing (Active Motif) according to the manufacturer's instructions. In brief, the soluble chromatin extracts were prepared from 2×10^8^ HEK293 cells, and immunoprecipitated with anti-T7 monoclonal antibodies and protein G magnetic beads (VERITAS) overnight. The immunoprecipitated DNA-histone complexes were incubated overnight at 65°C to reverse cross-linking and then treated with RNase A and protease K. Purified DNA fragments were subjected to quantitative PCR.

### Gel-retardation assays

Transfected COS-7 cells were lysed in lysis buffer containing 20 mM HEPES (pH 7.4), 150 mM NaCl, 12.5 mM β-glycerophosphate, 1.5 mM MgCl_2_, 2 mM EGTA, 10 mM NaF, 2 mM dithiothreitol, 1 mM Na_3_VO_4_, 1 mM phenylmethylsulfonyl fluoride, 100 units/ml aprotinin, 0.5% Triton X-100. Binding reactions were performed at room temperature for 30 minutes by incubating cell extracts and Cy5.5-labeled retardation probes in binding buffer containing 25 mM Tris (pH7.9), 250 mM KCl, 1 mM EDTA, 5% glycerol, 1 mM dithiothreitol, 0.25 mg/ml BSA, 0.1% Triton X-100 and 0.1 µg/ml of poly(dI)•poly(dC). The samples were analyzed on 3–12% polyacrylamide gels. For supershift experiments, anti-T7 antibodies or normal mouse IgG (Santa Cruz) (1 µg per lane) were added in the binding reactions. The sequences of the gel retardation probes are as follows: TRE2 probe, 5′-AATTGCTGAGTCACAGACAT-3′; mutated TRE2 probe, 5′-AATTGCAAGCTTACAGACAT-3′; probe deleting the core 6 bases of TRE2, 5′-AAATAATTGCCAGACATTAC-3′. TRE2 and mutated TRE2 are underlined.

### Yeast two-hybrid screening

The LexA DBD-KGB-1 (K67R) plasmid was used as bait to screen the *Caenorhabditis elegans* cDNA library in pACTII [38]. The bait plasmid and the library cDNAs were co-transformed into the *Saccharomyces cerevisiae* reporter strain L40 [*MAT*a, *trp1*, *leu2*, *his3*, *LYS2*::(*lexAop*)_4_-*HIS3*, *URA3*::(*lexAop*)_8_-*LacZ*]. Yeast cells were plated onto a synthetic medium plate lacking histidine and containing 3-amino triazole, and allowed to grow at 30°C. Transformants grown on selective medium plates were then streaked on selective medium plates again. Plasmids were collected from colonies that grew on selective medium plates and subjected to DNA sequencing.

## Supporting Information

Figure S1 FOS-1 is phosphorylated by KGB-1. (A) Phosphorylation of FOS-1 by KGB-1. COS-7 cells were co-transfected with expression vectors encoding T7-FOS-1, HA-KGB-1, and FLAG-MEK-1 as indicated. Whole cell extracts were analyzed by Western blot. (B) Effect of the T304E mutation on FOS-1. COS-7 cells were transfected with expression vectors encoding T7-FOS-1 WT and FOS-1(T304E) as indicated. Whole cell extracts were analyzed by Western blot.
(TIF)

Figure S2 Sites of FOS-1 phosphorylation by KGB-1. (A, B) COS-7 cells were co-transfected with expression vectors encoding T7-FOS-1 variants, HA-KGB-1 WT, HA-KGB-1 KN, and FLAG-MEK-1 as indicated. Whole cell extracts were analyzed by Western blot. In the FOS-1 variants, each Ser or Thr residue was replaced with Ala.
(TIF)

Figure S3 Effect of *fos-1* inhibition on stress sensitivity. Each animal was cultured from embryogenesis on normal plates containing copper sulfate (40 µM) and seeded with a bacteria strain expressing the double-stranded RNA for *fos-1*. The percentages of worms reaching adulthood 4 days after egg laying are shown with standard errors. Error bars indicate 95% confidence interval. **P<0.01 as determined by Student's *t* test. NS, not significant.
(TIF)

Figure S4 Identification of *kreg* genes. (A) Flow chart for microarray screening. Comparisons among groups subjected to different treatments are presented. There were 334 genes whose expression was up-regulated >2-fold between Cu^2+^-treated/non-treated wild-type animals. Of these 334 genes, 66 genes showed >2-fold up-regulation in Cu^2+^-treated wild-type animals/Cu^2+^-treated *kgb-1* animals. Of these 66 genes, 50 showed increase or no change in non-treated wild-type animals/non-treated *kgb-1* animals. (B) qRT-PCR analysis of genes isolated from microarray screen. Wild-type and *kgb-1* mutant animals were cultured on plates seeded with a bacteria strain. At 3 days after hatching, animals were treated with copper sulfate (1 mM) for 1 hour and total RNA was isolated. Expression of genes was analyzed by qRT-PCR and six genes were identified as *kreg* (*KGB-1-regulated gene*). Data are compared using a one-way ANOVA. *P<0.05, **P<0.01. NS, not significant.
(TIF)

Figure S5 Heavy metal sensitivity caused by inhibition of *kreg* genes. The *eri-1* mutant animals were cultured from embryogenesis on normal plates containing copper sulfate (100 µM) and seeded with bacteria strains expressing the indicated double-stranded RNA. The percentages of worms reaching adulthood 4 days after egg laying are shown with standard errors. Error bars indicate 95% confidence interval. **P<0.01 as determined by Student's *t* test. NS, not significant.
(TIF)

Figure S6 FOS-1 represses *kreg* expression. Wild-type and *kgb-1* mutant animals were cultured on plates seeded with a bacteria strain expressing the double-stranded RNA for *fos-1*. Total RNA was isolated and expression of *kreg-1* (A) and *kreg-2* (B) was analyzed by qRT-PCR. Data are compared using a one-way ANOVA. **P<0.01.
(TIF)

Figure S7 Expression of *mek-1* in the intestine determines resistance to heavy metal stress. Each animal was cultured from embryogenesis on normal plates containing copper sulfate (100 µM). The relative viability is shown with standard errors. Error bars indicate 95% confidence interval. **P<0.01 as determined by Student's *t* test.
(TIF)

Figure S8 Effects of JUN-1 and ATF-7 on the KGB-1 pathway. (A) Effect of JUN-1 and ATF-7 on *kreg-1* expression. Wild-type animals harboring the *Pkreg-1::venus* transgene as an extrachromosomal array were cultured on plates seeded with a bacteria strain expressing the double-stranded RNA for *fos-1*, *jun-1* or *atf-7*. “Weak” refers to animals in which intestinal Venus was present at low levels. “Strong” indicates that Venus was present at high levels in most of the intestine. Percentages of animals in each expression category are listed. The numbers (n) of animals examined are shown. (B) Effect of JUN-1 on heavy metal sensitivity. Each animal was cultured from embryogenesis on normal plates containing copper sulfate (40 µM) and seeded with a bacteria strain expressing the double-stranded RNA for *jun-1*. The percentages of worms reaching adulthood 4 days after egg laying are shown with standard errors. Error bars indicate 95% confidence interval. **P<0.01 as determined by Student's *t* test. NS, not significant. (C) Effect of ATF-7 on heavy metal sensitivity. Each animal was cultured from embryogenesis on normal plates containing copper sulfate (40 µM). The percentages of worms reaching adulthood 4 days after egg laying are shown with standard errors.
(TIF)

Figure S9 Effect of *hda-1* depletion on expression of *kreg-1* and *kreg-2* genes. Wild-type and *kgb-1* mutant animals were cultured on plates seeded with a bacteria strain expressing the double-stranded RNA for *hda-1*. Total RNA was isolated and expression of *kreg-1* (A) and *kreg-2* (B) was analyzed by qRT-PCR. Data are compared using a one-way ANOVA. **P<0.01.
(TIF)

Table S1 List of KGB-1 interacting proteins isolated by yeast two-hybrid screening. A *C. elegans* mixed-stage cDNA library were screened by the yeast two-hybrid method to isolate proteins that interact with KGB-1. From this screen, we identified 10 proteins that interact with KGB-1.
(XLS)

Table S2 Comparison of transcript expression levels between wild-type animals with or without Cu^2+^ treatment. A microarray analysis was carried out to examine gene expression changes in wild-type animals subjected to heavy metal stress. Transcript expression levels were compared between wild-type animals with or without Cu^2+^ treatment.
(XLSX)

Table S3 Comparison of transcript expression levels between *kgb-1* mutant animals with or without Cu^2+^ treatment. A microarray analysis was carried out to examine gene expression changes in *kgb-1* mutant animals subjected to heavy metal stress. Transcript expression levels were compared between *kgb-1* mutant animals with or without Cu^2+^ treatment.
(XLSX)

Table S4 List of genes that were up-regulated by Cu^2+^ in wild-type animals. A microarray analysis was carried out to examine gene expression changes in wild-type and *kgb-1* mutant animals subjected to heavy metal stress. 334 genes were chosen that were up-regulated greater than 2-fold by Cu^2+^ in wild-type animals.
(XLSX)

Table S5 List of genes whose induction by Cu^2+^ in *kgb-1* mutants was <50% of the induction seen in wild-type animals. Among the 334 genes listed in Table S4, we identified 66 genes whose induction by Cu^2+^ in *kgb-1* mutants was <50% of the induction seen in wild-type animals.
(XLSX)

Table S6 List of genes whose basal expression was decreased or not changed in *kgb-1* mutants versus wild-type animals. Among the 66 genes listed in Table S5, we identified 50 genes whose basal expression was decreased or not changed in *kgb-1* mutants versus wild-type animals.
(XLSX)

Table S7 List of the top thirteen genes whose expression was significantly induced by Cu^2+^ in wild-type animals. Among the 50 genes listed in Table S6, we manually removed genes no longer predicted to be expressed, and selected the top 13 genes whose expression was significantly induced by Cu^2+^ in wild-type animals.
(XLSX)

Table S8 List of six *kreg* genes. We analyzed the 13 genes listed in Table S7 by qRT-PCR and confirmed that six of these are genes whose expression is regulated by KGB-1. These were designated as *kreg* genes (*KGB-1-regulated gene*).
(XLSX)
