# Supplementary figures and images for: The Caenorhabditis elegans JNK Signaling Pathway Activates Expression of Stress Response Genes by Derepressing the Fos/HDAC Repressor Complex
Source: PLoS Genet. 2013 Feb 21;9(2):e1003315. doi: 10.1371/journal.pgen.1003315 (PMC3578760; doi:10.1371/journal.pgen.1003315)

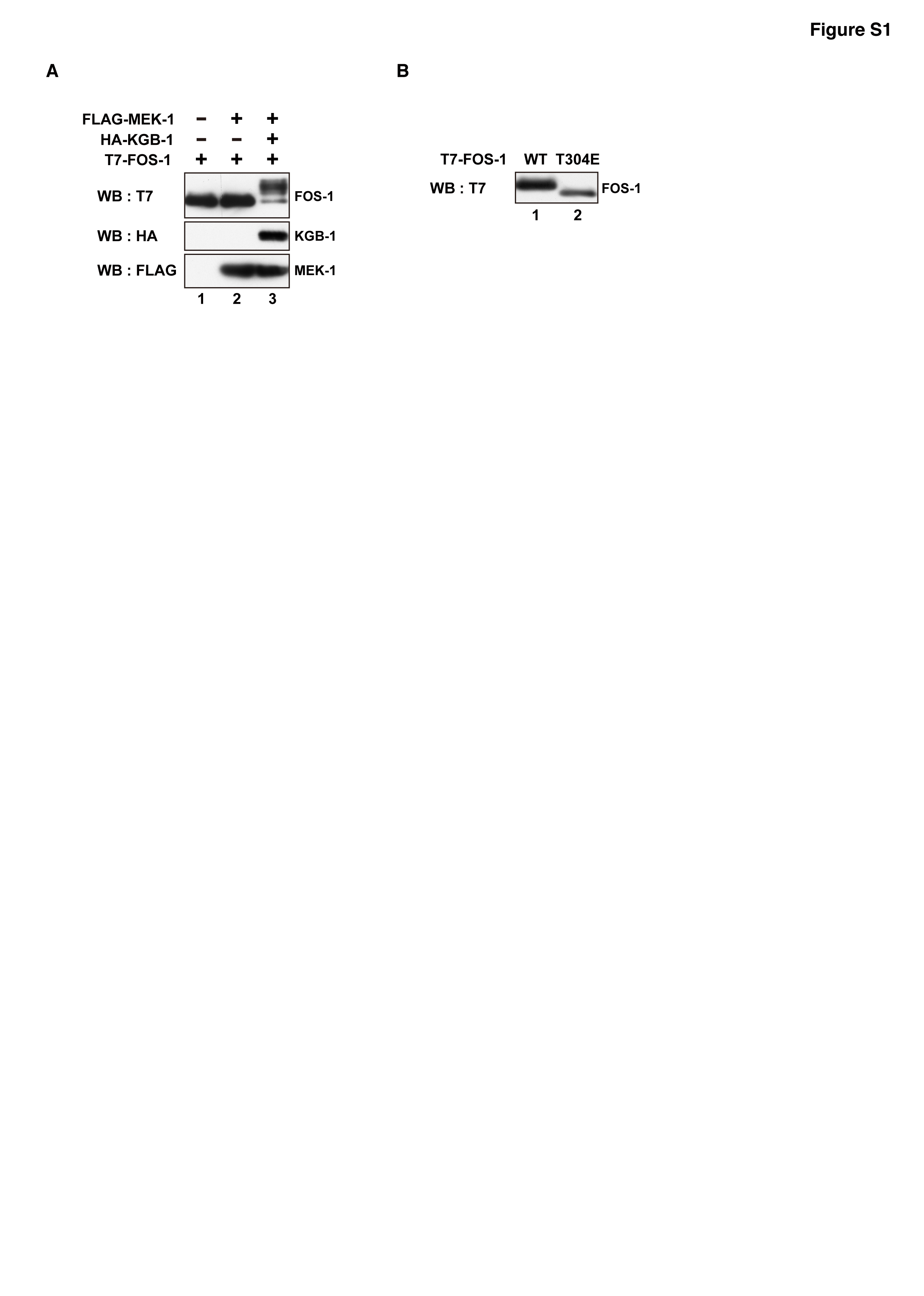

Supplement: Figure S1 — FOS-1 is phosphorylated by KGB-1. (A) Phosphorylation of FOS-1 by KGB-1. COS-7 cells were co-transfected with expression vectors encoding T7-FOS-1, HA-KGB-1, and FLAG-MEK-1 as indicated. Whole cell extracts were analyzed by Western blot. (B) Effect of the T304E mutation on FOS-1. COS-7 cells were transfected with expression vectors encoding T7-FOS-1 WT and FOS-1(T304E) as indicated. Whole cell extracts were analyzed by Western blot. (TIF) [file pgen.1003315.s001.tif]

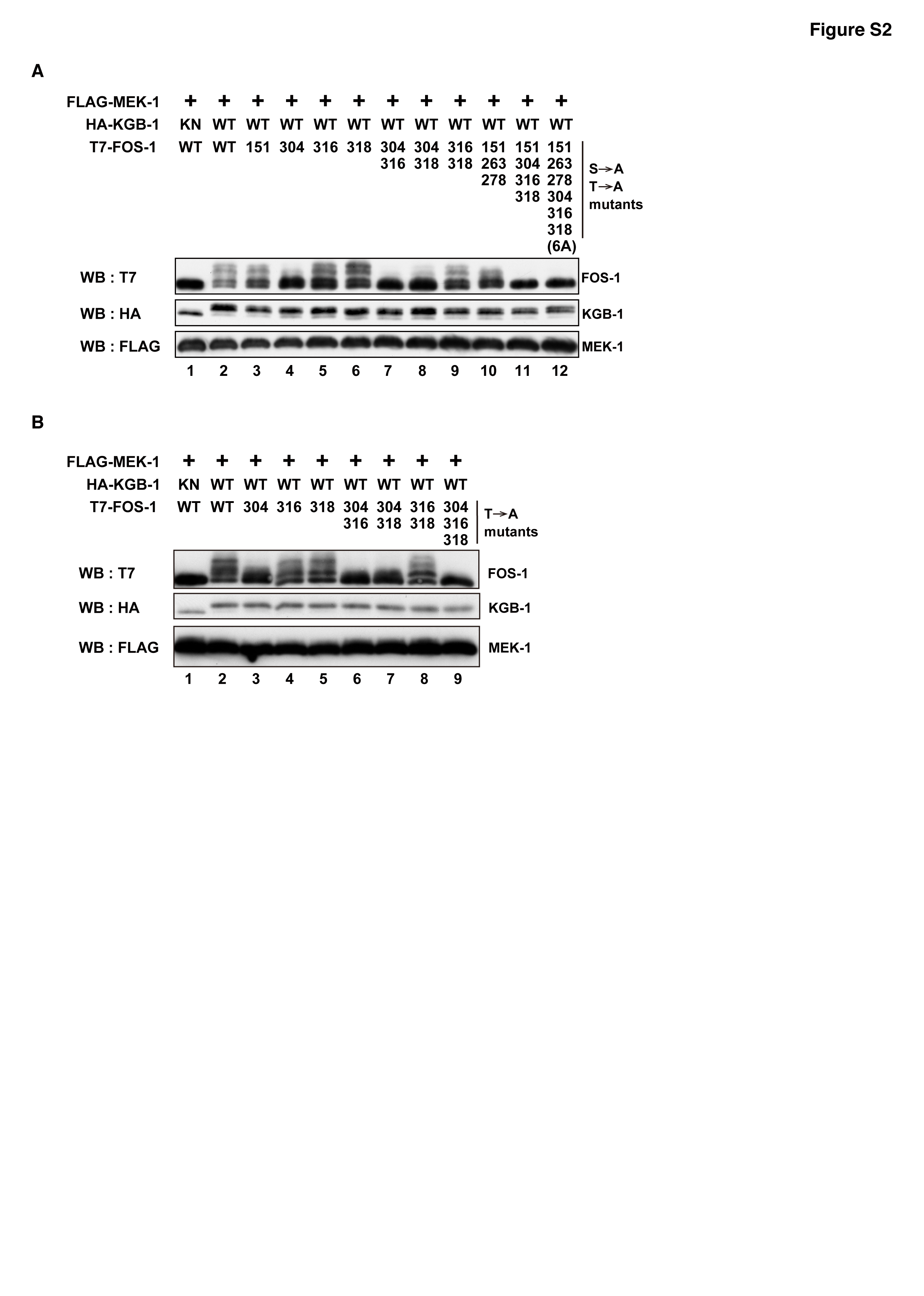

Supplement: Figure S2 — Sites of FOS-1 phosphorylation by KGB-1. (A, B) COS-7 cells were co-transfected with expression vectors encoding T7-FOS-1 variants, HA-KGB-1 WT, HA-KGB-1 KN, and FLAG-MEK-1 as indicated. Whole cell extracts were analyzed by Western blot. In the FOS-1 variants, each Ser or Thr residue was replaced with Ala. (TIF) [file pgen.1003315.s002.tif]

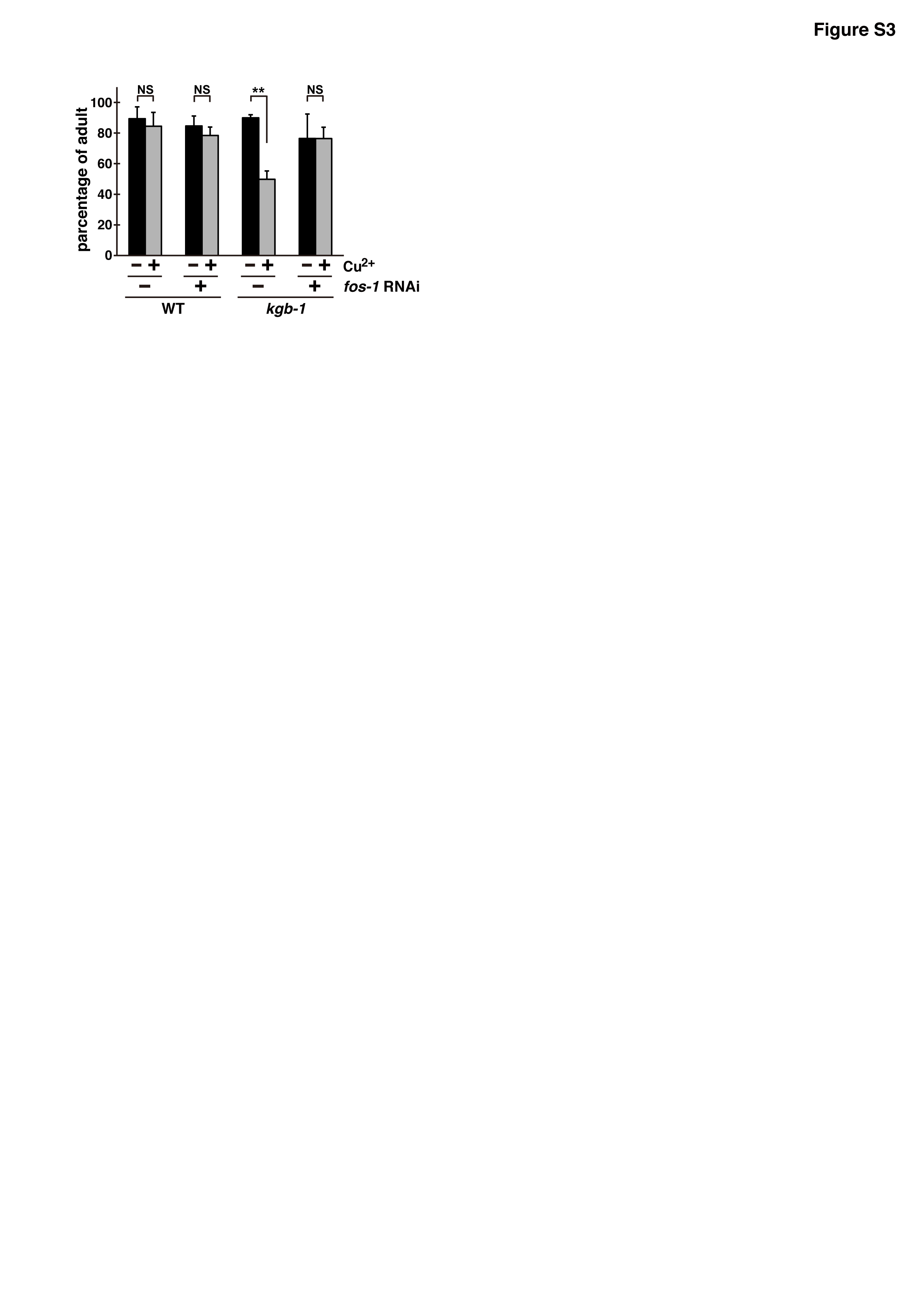

Supplement: Figure S3 — Effect of fos-1 inhibition on stress sensitivity. Each animal was cultured from embryogenesis on normal plates containing copper sulfate (40 µM) and seeded with a bacteria strain expressing the double-stranded RNA for fos-1. The percentages of worms reaching adulthood 4 days after egg laying are shown with standard errors. Error bars indicate 95% confidence interval. **P<0.01 as determined by Student's t test. NS, not significant. (TIF) [file pgen.1003315.s003.tif]

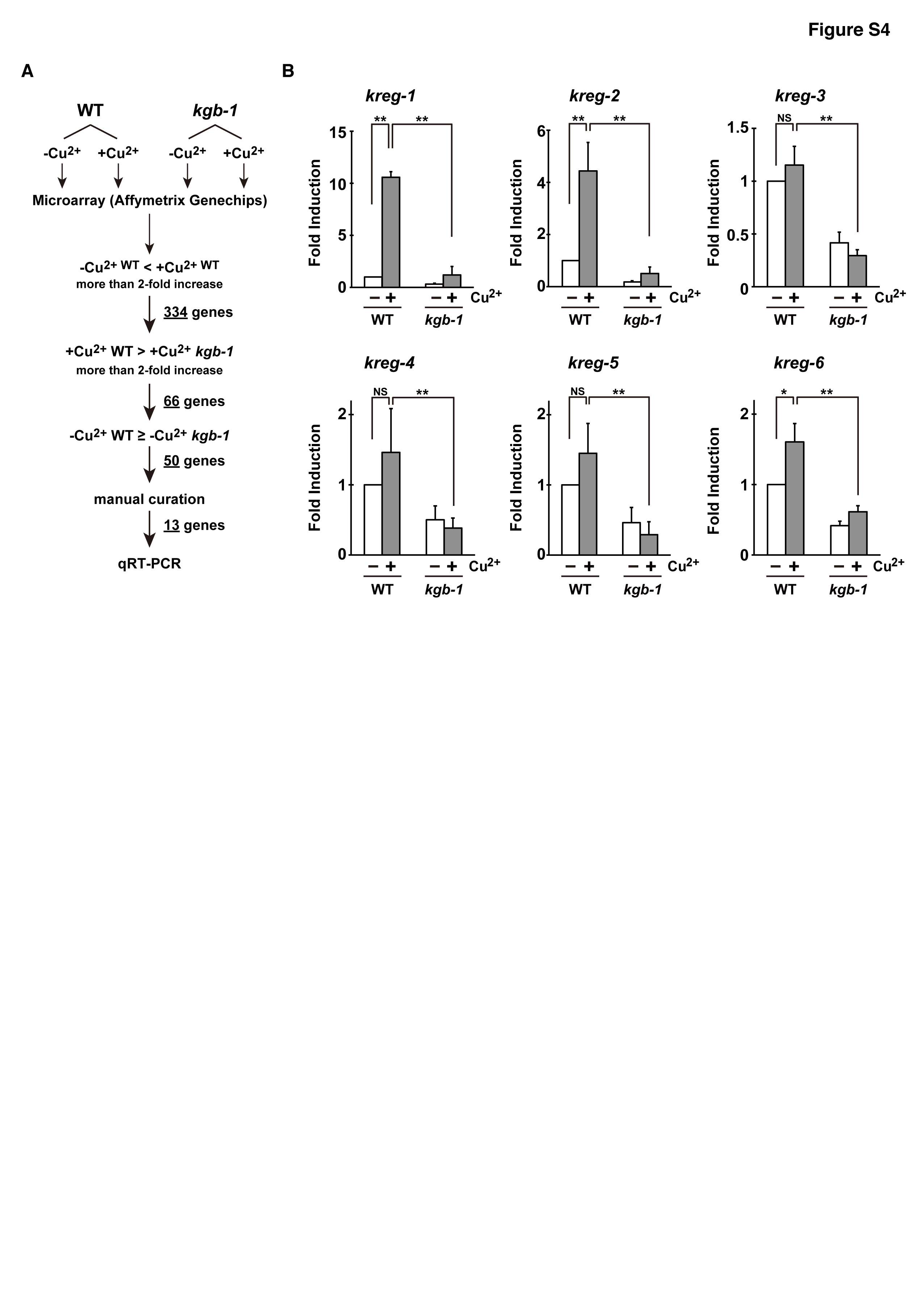

Supplement: Figure S4 — Identification of kreg genes. (A) Flow chart for microarray screening. Comparisons among groups subjected to different treatments are presented. There were 334 genes whose expression was up-regulated >2-fold between Cu2+-treated/non-treated wild-type animals. Of these 334 genes, 66 genes showed >2-fold up-regulation in Cu2+-treated wild-type animals/Cu2+-treated kgb-1 animals. Of these 66 genes, 50 showed increase or no change in non-treated wild-type animals/non-treated kgb-1 animals. (B) qRT-PCR analysis of genes isolated from microarray screen. Wild-type and kgb-1 mutant animals were cultured on plates seeded with a bacteria strain. At 3 days after hatching, animals were treated with copper sulfate (1 mM) for 1 hour and total RNA was isolated. Expression of genes was analyzed by qRT-PCR and six genes were identified as kreg (KGB-1-regulated gene). Data are compared using a one-way ANOVA. *P<0.05, **P<0.01. NS, not significant. (TIF) [file pgen.1003315.s004.tif]

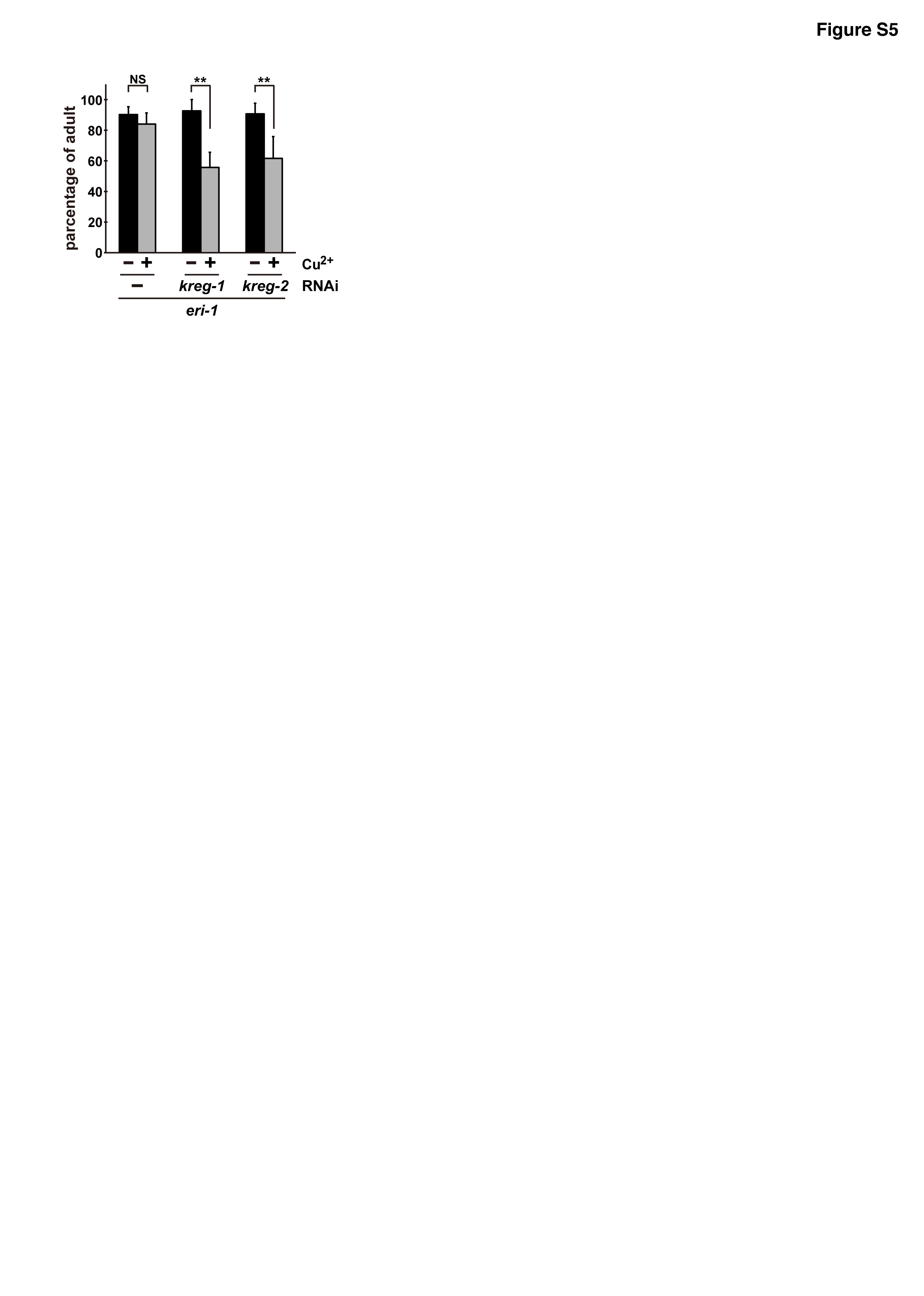

Supplement: Figure S5 — Heavy metal sensitivity caused by inhibition of kreg genes. The eri-1 mutant animals were cultured from embryogenesis on normal plates containing copper sulfate (100 µM) and seeded with bacteria strains expressing the indicated double-stranded RNA. The percentages of worms reaching adulthood 4 days after egg laying are shown with standard errors. Error bars indicate 95% confidence interval. **P<0.01 as determined by Student's t test. NS, not significant. (TIF) [file pgen.1003315.s005.tif]

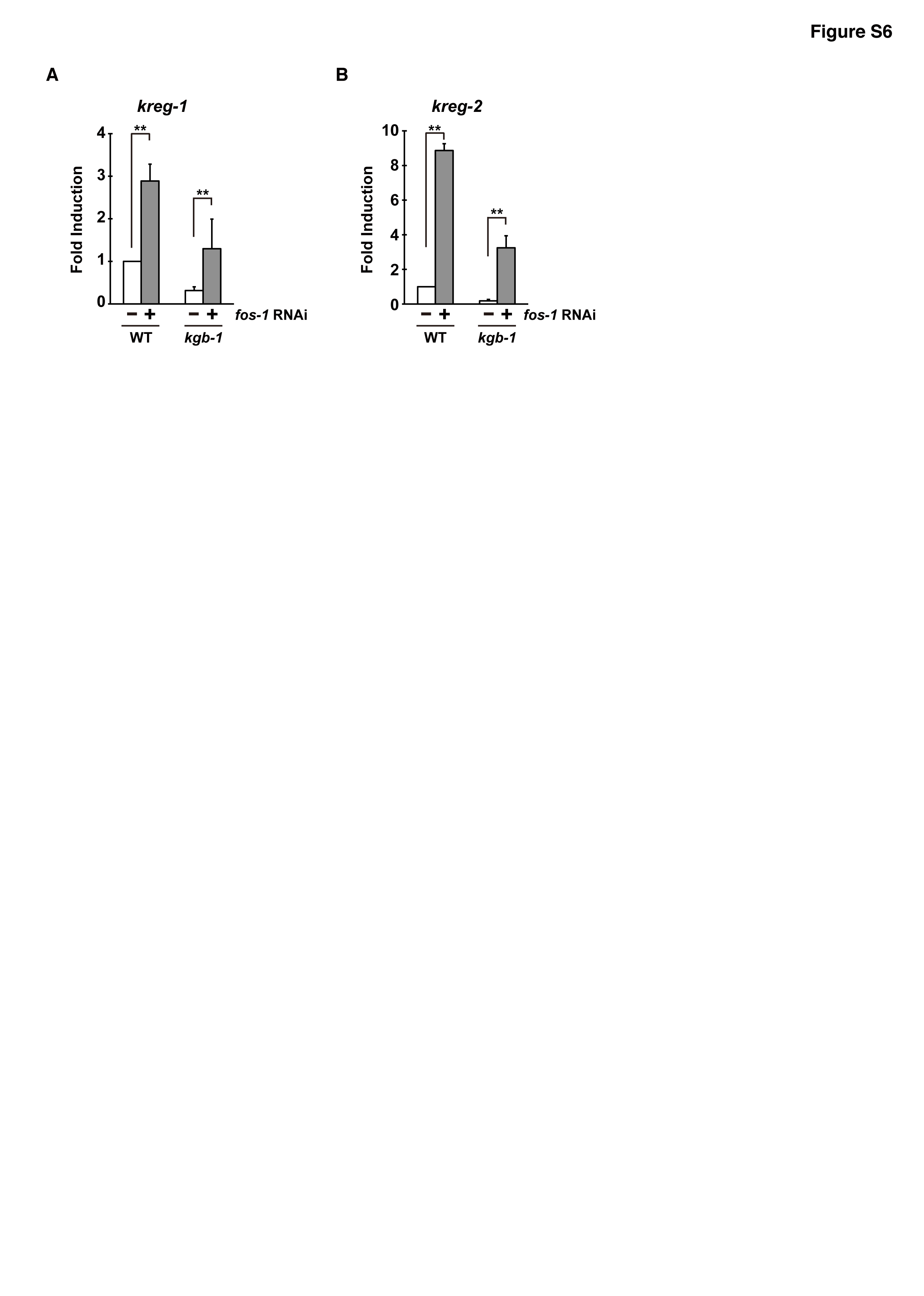

Supplement: Figure S6 — FOS-1 represses kreg expression. Wild-type and kgb-1 mutant animals were cultured on plates seeded with a bacteria strain expressing the double-stranded RNA for fos-1. Total RNA was isolated and expression of kreg-1 (A) and kreg-2 (B) was analyzed by qRT-PCR. Data are compared using a one-way ANOVA. **P<0.01. (TIF) [file pgen.1003315.s006.tif]

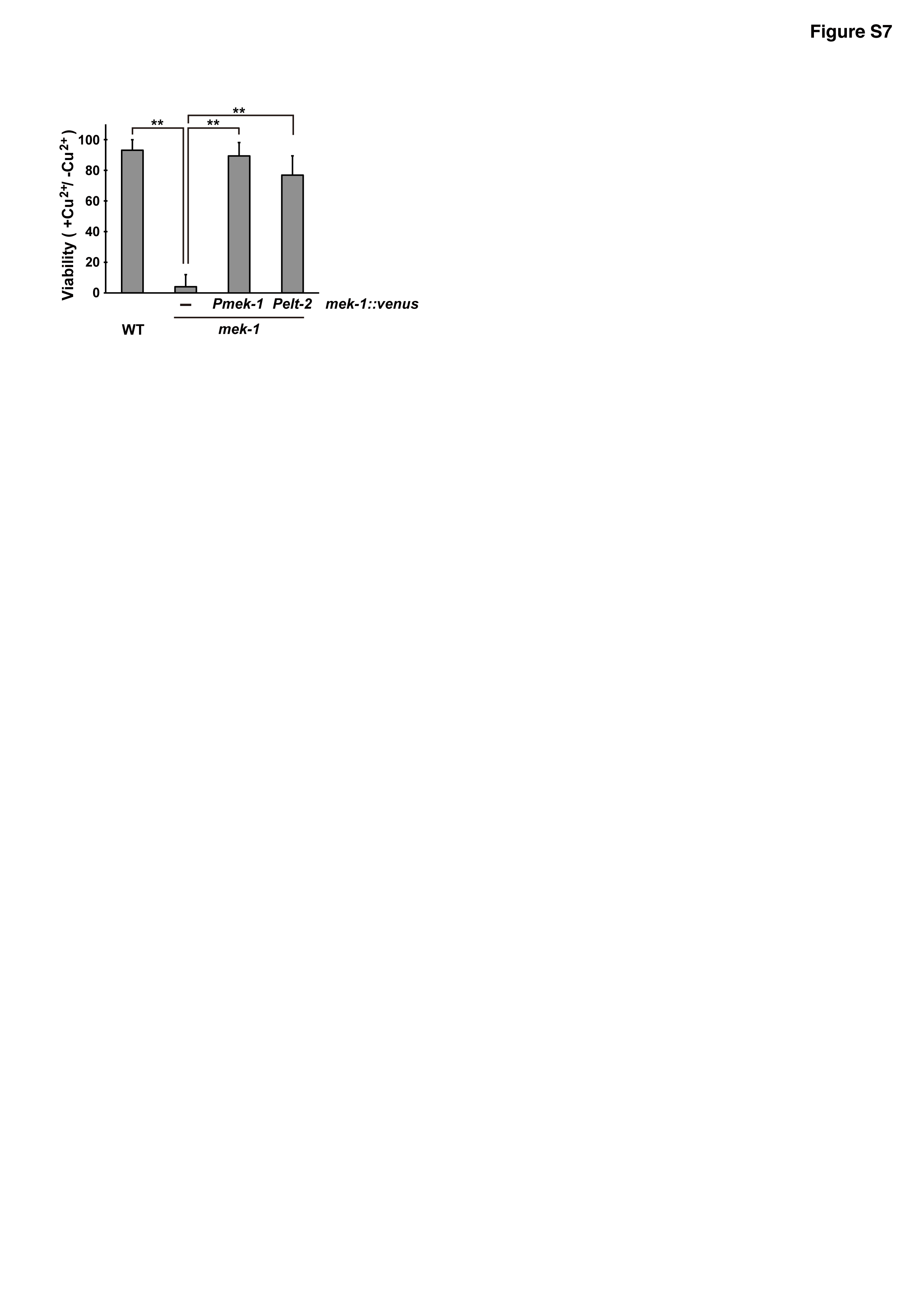

Supplement: Figure S7 — Expression of mek-1 in the intestine determines resistance to heavy metal stress. Each animal was cultured from embryogenesis on normal plates containing copper sulfate (100 µM). The relative viability is shown with standard errors. Error bars indicate 95% confidence interval. **P<0.01 as determined by Student's t test. (TIF) [file pgen.1003315.s007.tif]

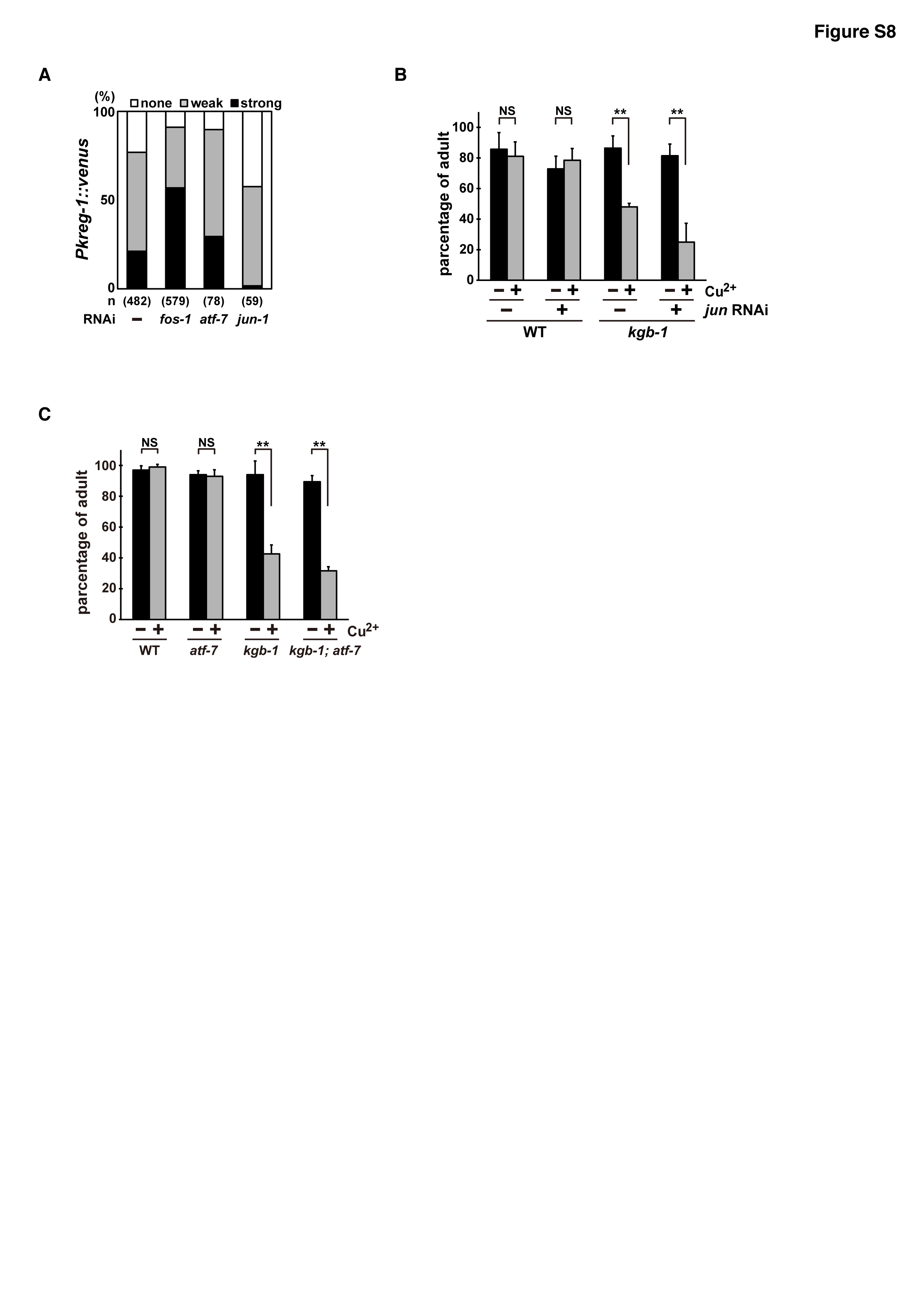

Supplement: Figure S8 — Effects of JUN-1 and ATF-7 on the KGB-1 pathway. (A) Effect of JUN-1 and ATF-7 on kreg-1 expression. Wild-type animals harboring the Pkreg-1::venus transgene as an extrachromosomal array were cultured on plates seeded with a bacteria strain expressing the double-stranded RNA for fos-1, jun-1 or atf-7. “Weak” refers to animals in which intestinal Venus was present at low levels. “Strong” indicates that Venus was present at high levels in most of the intestine. Percentages of animals in each expression category are listed. The numbers (n) of animals examined are shown. (B) Effect of JUN-1 on heavy metal sensitivity. Each animal was cultured from embryogenesis on normal plates containing copper sulfate (40 µM) and seeded with a bacteria strain expressing the double-stranded RNA for jun-1. The percentages of worms reaching adulthood 4 days after egg laying are shown with standard errors. Error bars indicate 95% confidence interval. **P<0.01 as determined by Student's t test. NS, not significant. (C) Effect of ATF-7 on heavy metal sensitivity. Each animal was cultured from embryogenesis on normal plates containing copper sulfate (40 µM). The percentages of worms reaching adulthood 4 days after egg laying are shown with standard errors. (TIF) [file pgen.1003315.s008.tif]

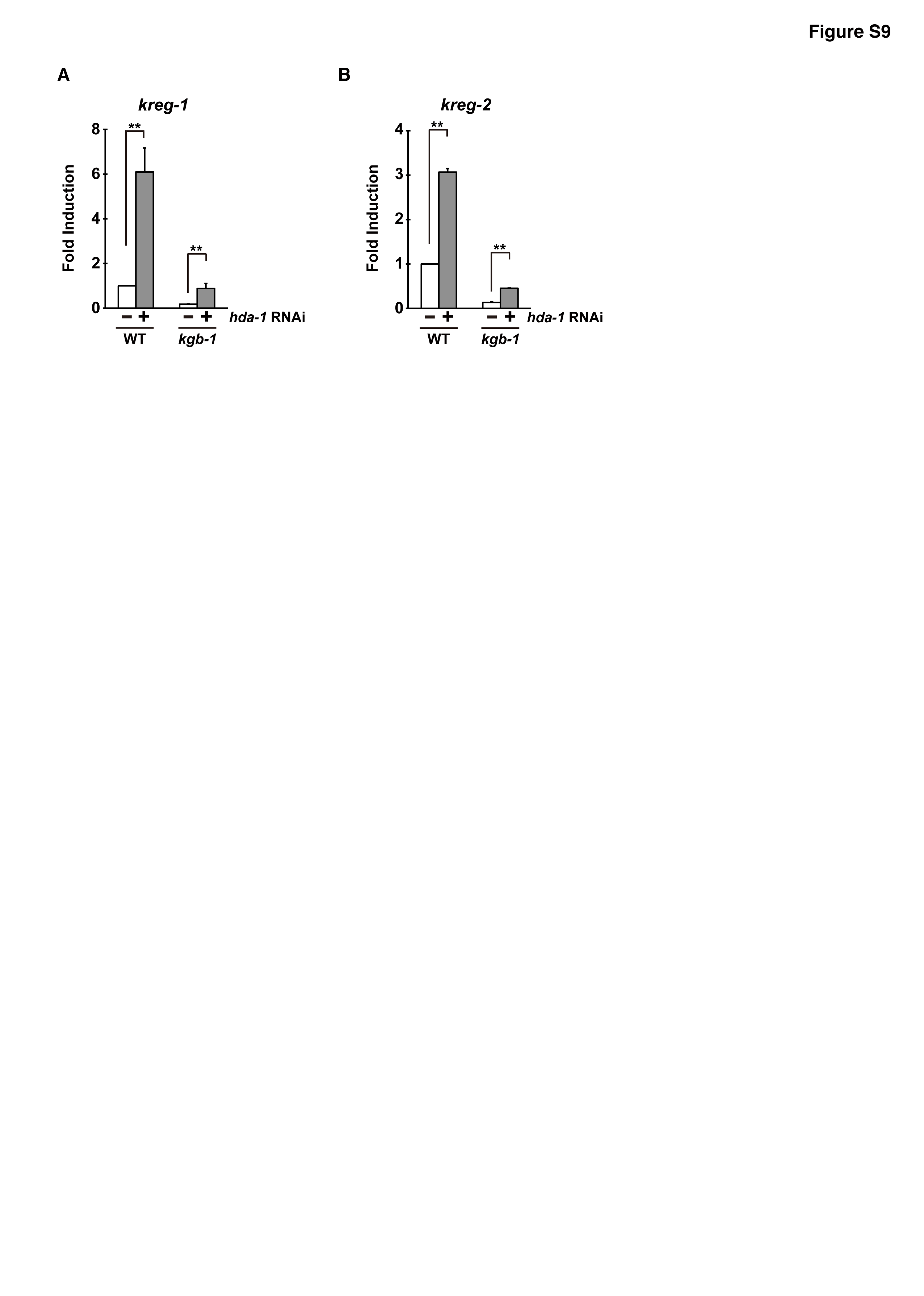

Supplement: Figure S9 — Effect of hda-1 depletion on expression of kreg-1 and kreg-2 genes. Wild-type and kgb-1 mutant animals were cultured on plates seeded with a bacteria strain expressing the double-stranded RNA for hda-1. Total RNA was isolated and expression of kreg-1 (A) and kreg-2 (B) was analyzed by qRT-PCR. Data are compared using a one-way ANOVA. **P<0.01. (TIF) [file pgen.1003315.s009.tif]
